# Therapeutic potential of TRPM8 antagonists in prostate cancer

**DOI:** 10.1038/s41598-021-02675-4

**Published:** 2021-12-01

**Authors:** Marzia Di Donato, Carmine Ostacolo, Pia Giovannelli, Veronica Di Sarno, Isabel M. Gomez Monterrey, Pietro Campiglia, Antimo Migliaccio, Alessia Bertamino, Gabriella Castoria

**Affiliations:** 1 Department of Precision Medicine, School of Medicine, University of Campania ‘L. Vanvitelli’, Via L. De Crecchio 7, 80138 Naples, Italy; 2 Department of Pharmacy, University Federico II of Naples, Via D. Montesano 49, 80131 Naples, Italy; 3 Department of Pharmacy, University of Salerno, Via G.Paolo II, 84084 Fisciano, SA Italy

**Keywords:** Prostate cancer, Medicinal chemistry

## Abstract

Transient receptor potential melastatin-8 (TRPM8) represents an emerging target in prostate cancer, although its mechanism of action remains unclear. Here, we have characterized and investigated the effects of TRPM8 modulators in prostate cancer aggressiveness disclosing the molecular mechanism underlying their biological activity. Patch-clamp and calcium fluorometric assays were used to characterize the synthesized compounds. Androgen-stimulated prostate cancer-derived cells were challenged with the compounds and the DNA synthesis was investigated in a preliminary screening. The most effective compounds were then employed to inhibit the pro-metastatic behavior of in various PC-derived cells, at different degree of malignancy. The effect of the compounds was then assayed in prostate cancer cell-derived 3D model and the molecular targets of selected compounds were lastly identified using transcriptional and non-transcriptional reporter assays. TRPM8 antagonists inhibit the androgen-dependent prostate cancer cell proliferation, migration and invasiveness. They are highly effective in reverting the androgen-induced increase in prostate cancer cell spheroid size. The compounds also revert the proliferation of castrate-resistant prostate cancer cells, provided they express the androgen receptor. In contrast, no effects were recorded in prostate cancer cells devoid of the receptor. Selected antagonists interfere in non-genomic androgen action and abolish the androgen-induced androgen receptor/TRPM8 complex assembly as well as the increase in intracellular calcium levels in prostate cancer cells. Our results shed light in the processes controlling prostate cancer progression and make the transient receptor potential melastatin-8 as a ‘*druggable*’ target in the androgen receptor-expressing prostate cancers.

## Introduction

The transient receptor potential melastatin-8 (TRPM8), a widely distributed non-selective calcium permeable ion channel, was initially characterized as a cold sensing channel^1,2^. It is involved in a plethora of pathological processes, such as pain^3,4^, migraine^5^, overactive as well as painful bladder syndromes^6,7^ and dry eye syndrome or excessive lacrimation dysfunction^8^. TRPM8 channel is directly involved in calcium homeostasis^9^ and can be regulated by androgens^10^, estrogens^11^, neurotrophins^12^ and growth factors^13^. As such, it represents a very promising target in solid cancers^13,14^, including prostate cancer (PC)^9,15^.

PC is the second most frequently diagnosed cancer in men and several therapeutic approaches are currently available for patient’s care. Nevertheless, PC might escape the treatments, giving rise to more aggressive forms of cancer. At this stage, PC often spreads, with a significant increase in the mortality rate^16^. Therefore, new approaches are needed for a better pharmacological management in PC patients with advanced disease.

TRPM8 gene was firstly identified as a reporter of the androgen receptor (AR) transcriptional activity in PC cells^17^ and subsequently proposed as a PC biomarker. Its expression, indeed, increases during the initial stages of the disease, to decline after androgen deprivation therapy^18^. Although TRPM8 expression depends on AR transcriptional activity^19^, biochemical findings have reported a direct TRPM8 interaction with androgens^20^ or the androgen receptor (AR)^21^. Nowadays, regulation of TRPM8 by the androgen/AR axis remains debated, although pharmacologic modulation of the channel is frequently proposed in PC therapy^20,22–24^.

Our groups investigated the rapid androgen action in quite divergent cell types^25–28^ and designed several TRPM8 modulators, characterized by selectivity towards other TRP channels, the highest ‘in vitro’ potency and metabolic stability so far described^29–32^.

Here, we report the effect of selected TRPM8 modulators and their analogues in various PC-derived cell lines, including LNCaP cells that represent a suitable model to investigate the role of sex steroids in PC-derived cells^33^. TRPM8 antagonists reverse the increase in calcium influx and the rapid signaling activation leading to the androgen-elicited invasion and proliferation in various PC-derived cells as well as to the increase in LNCaP spheroid size. These findings, together with the observation the TRPM8 modulators inhibit the proliferation of AR-expressing castrate resistant prostate cancer (CRPC) cells, while leaving unaffected the behavior of AR-negative PC cells, offer new insights into the knowledge of TRPM8 in PC pathogenesis and pave the way for novel promising strategies in clinical management of PC patients.

## Results

### In vitro pharmacological characterization

The molecular structure of tested compounds is shown in Figure S1 (Supplementary Information). They were synthesized following the procedures described in Figure S2 (Supplementary Information). Potency of these compounds over TRPM8 channels was evaluated by patch-clamp assays. Compounds **3** and **6** have been previously tested for their agonist and antagonist effect, respectively, over TRPM8 (Table S1, Supplementary Information). Compound **3** showed a dose-dependent agonistic activity with higher potency than L-Menthol (EC_50_ = 40 μM)^30^, while compound **6** is the most potent in vitro TRPM8 antagonist described so far with an IC_50_ of 0.2 nM^29^. Both, agonist and antagonist were chosen as reference compounds to identify the type of TRPM8 modulator more suitable to produce an antiproliferative effect in PC cells. This matter is widely debated, since both TRPM8 agonist and antagonist have been proposed for PC treatment, albeit a different mechanism seems to be involved^9^.

In addition, three new analogs (**4**, **5**, and **9**) were synthesized and characterized by patch clamp electrophysiological assays. These chemotypes are particularly attractive due to the potent and selective cytotoxicity shown in a previous drug discovery screening^34^. Compounds **4** and **5** showed high potency as TRPM8 antagonist with a IC_50s_ in the high nanomolar range (204–340 nM, Table S1 and Figure S3 in Supplementary Information). The inhibitory effects of both compounds on menthol-evoked TRPM8 currents were concentration-dependent (Figure S3 in Supplementary Information). Indeed, compound **9** proved to be an effective concentration-dependent TRPM8 antagonist although less potent, with an IC_50_ in the low micromolar range (5.0 mM, Table S1 and Figure S3 in Supplementary Information). All the compounds behaved as reversible inhibitor, since menthol-evoked currents were fully recovered during the drug’s washout.

The typical drawback of TRPM8 modulators is represented by the lack of selectivity over other TRP channels, particularly TRPA1 and TRPV1. Indeed, the same prototypical TRPM8 agonists (menthol) and antagonists (AMTB), modulate the activity of different TRP channels subtypes^35,36^. To address this issue, the selectivity of synthesized compounds was challenged by calcium fluorometric assays. Compounds **3** and **6** selectivity has been previously assessed^29,30^. Accordingly, the newly synthesized compounds showed no agonistic or antagonistic effect when tested in cell lines expressing TRPA1 or TRPV1 channels (Figure S4 in Supplementary Information). Furthermore, the sodium channel Nav1.7 is involved in PC cell invasiveness^37^. To rule out the effect of synthesized compounds over this channel, calcium fluorometric assays using HEK-293 stably expressing the human isoform of Nav1.7 channel were performed. As in the previous assays, tested compounds were unable to act as agonist or antagonist of this channel (Figure S4 in Supplementary Information).

### TRPM8 modulators inhibit the DNA synthesis and cell-cycle progression induced by androgens in various AR-expressing PC cells

**Figure 1:**
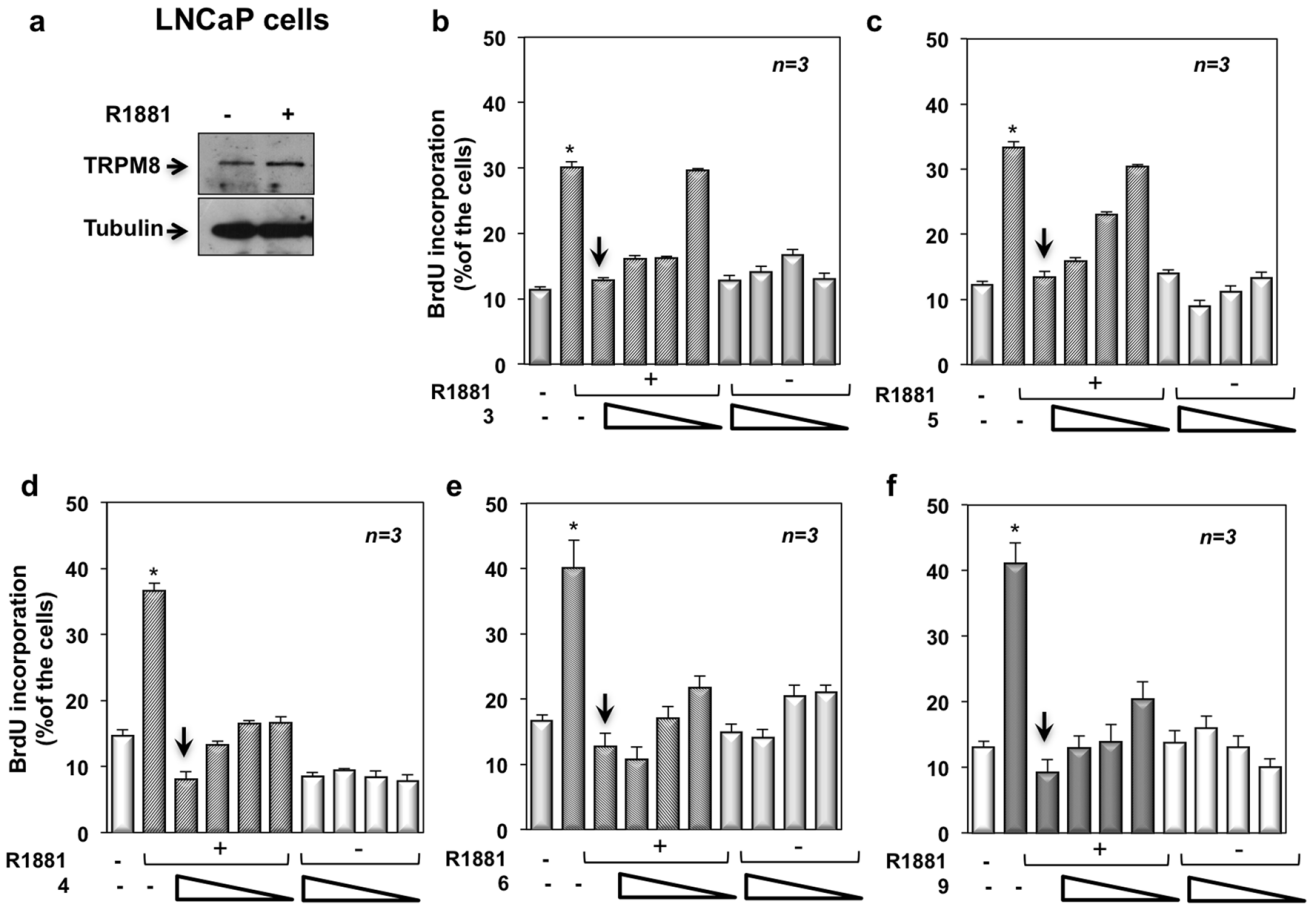
Effect of TRPM8 modulators on DNA synthesis elicited by androgens in PC-derived LNCaP cells. LNCaP cells were used. In (**a**), lysate protein from cells unstimulated (–) or stimulated for 24 h with 10 nM R1881 were analyzed by WB technique, using the antibodies against the indicated proteins. WB are representative of three different experiments and densitometric analysis of data is reported in Supplementary Information. In (**b**–**f**) quiescent LNCaP cells on coverslips were left untreated or treated for 18 h with 10 nM R1881, in the absence or presence of decreasing (1 μM, 100 nM, 10 nM or 1 nM) of the indicated TRPM8 modulators. Cells were pulsed in vivo with 100 μM BrdU and its incorporation into newly synthesized DNA was analyzed by IF, as described in “Methods”. BrdU incorporation was expressed as % of total cells. Means and standard errors (SEMs) are shown. *n,* represents the number of experiments. *p < 0.05 for the indicated experimental points *vs.* the corresponding untreated control.

**Figure 2:**
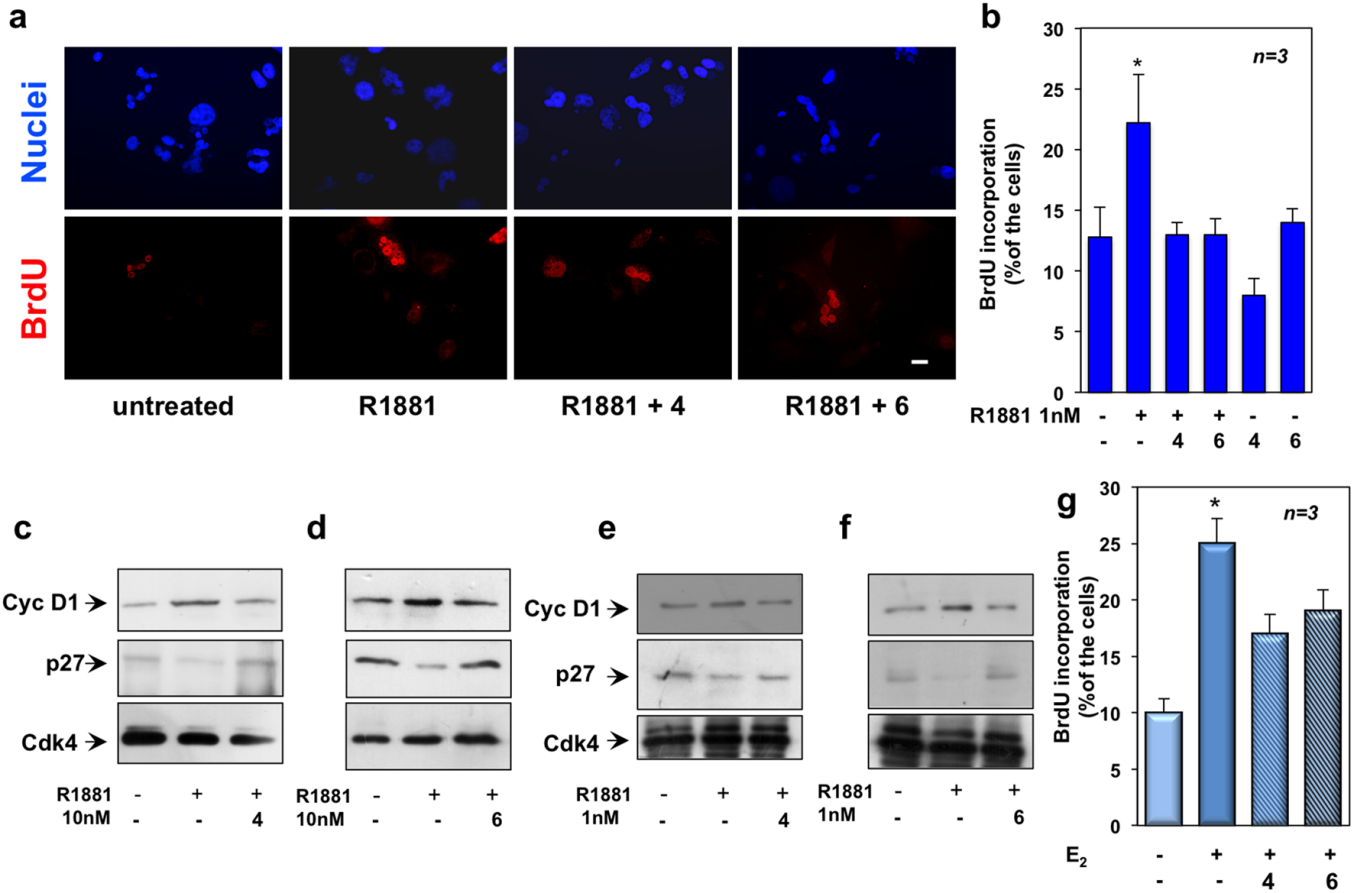
TRPM8 antagonists, 4 and 6, specifically affect the DNA synthesis and cell cycle progression elicited by androgens in LNCaP cells. LNCaP cells were employed. When indicated in Figure, the compounds **4** and **6** were used at 1 μM. Panel (**a**) shows representative images captured from one experiment presented in Fig. 1 (panels **d**,**e**). Fluorescence (red) in the lower section is from reactivity with the anti-BrdU antibody. Hoechst 33,258 staining (blue) is shown in the upper section. Bar, 10 μM. In (**b**), quiescent cells on coverslips were left untreated or treated for 18 h with 1 nM R1881 in the absence or presence of the indicated compounds. Cells were pulsed in vivo with 100 μM BrdU and its incorporation into DNA was analyzed and expressed as in Fig. 1. In (**c**–**f**), quiescent cells were left untreated or treated with 10 nM R1881 (**c**–**d**) or 1 nM R1881 (**e**–**f**) for 15 h, in the absence or presence of the indicated compounds. Lysate proteins were prepared and analyzed by WB technique, using the antibodies against the indicated proteins. WB are representative of three different experiments and the corresponding densitometric analysis is reported in Supplementary Information. In (**g**), quiescent cells on coverslips were left untreated or treated for 18 h with 10 nM estradiol (E_2_), in the absence or presence of the indicated compounds. Cells were pulsed in vivo with 100 μM BrdU and its incorporation into DNA was analyzed and expressed as in Fig. 1. In (**b**,**g**), means and standard errors (SEMs) are shown. *n,* represents the number of experiments. * *p* < 0.05 for the indicated experimental points *vs.* the corresponding untreated control.

We then used PC-derived LNCaP cells, which express AR and the isoform β of ER (Figure S5 in Supplementary Information) and respond to androgens and estrogens in terms of proliferation^28^. In a first attempt, we evaluated the expression of TRPM8 in these cells. Stimulation of quiescent LNCaP cells with 10 nM of the non-aromatizable androgen R1881 very weakly increased the amount of TRMP8, in the presence of similar amounts of tubulin, as assessed by Western blot analysis of lysate proteins (Fig. 1a). We next analyzed the effect of all the compounds on DNA synthesis induced by androgens in LNCaP cells. Quiescent cells were left untreated or treated with 10 nM R1881, in the absence or presence of increasing concentration (from 1 nM to 1 μM) of the indicated compounds. Almost all of them reversed the DNA synthesis stimulated by 10 nM R1881 in LNCaP cells (panels **b**–**f** in Fig. 1). The inhibitory effect observed by 1 μM of the TRPM8 antagonists **4** (Fig. 1d), or **6** (Fig. 1e) or **9** (Fig. 1f) was stronger than that observed using the same concentration of the agonist **3** (Fig. 1b) or antagonist **5** (Fig. 1c). All the tested compounds did not affect the DNA synthesis when added alone to LNCaP cells (panels **b**–**f** in Fig. 1). Representative IF images captured from one experiment in Fig. 1d,e shown in Fig. 2a. Of note, our results are consistent with the observation that WS-12, a potent and selective TRPM8 agonist, slightly modify the viability of LNCaP cells^15^, while antagonists exert a remarkable effect in the androgen-sensitive cell lines^23^. The higher efficacy of compounds **4**, **6** and **9**, as compared to the analogue **5**, might be due to their different potency and efficacy as TRPM8 antagonists (Table S1 in Supplementary Information).

Given the findings here presented, we investigated the compounds **4** and **6** throughout the manuscript (Table S1 in Supplementary Information).

One nM androgen concentration is optimal in BrdU incorporation studies reported in LNCaP cells^38^ and AR occupancy is almost completely saturated by that ligand concentration^39^. Therefore, we verified the effect of low ligand concentration in our assay. A slight (almost 2-folds), but significant increase of the DNA synthesis was recorded in LNCaP cells stimulated with 1 nM R1881, as compared with the more robust increase (almost 3,5-folds) in BrdU incorporation observed in cells stimulated with 10 nM R1881 (panels b–f in Fig. 1). These findings support a dose-dependent ligand effect. Noticeably, AR synthesis and degradation, the receptor’s amount, the affinity as well as on/off rates of AR ligand interactions might influence the AR ligand binding activity^39^. However, the compounds **4** and **6** also reverted the effect by 1 nM R1881 (Fig. 2b).

Androgens upregulates cyclin D1 and simultaneously downregulates p27 in LNCaP cells^28^. Ten nM R1881 up-regulated cyclin D1 in LNCaP cells (upper section in panels c and d of Fig. 2). A simultaneous decrease in p27 levels was observed (middle section in panels c and d of Fig. 2). Consistent with data on the DNA synthesis, 1 nM R1881 slightly increased cyclin D1 expression, simultaneously with a p27 decrease (Fig. 2e,f). Thus, androgen modulation of cell cycle markers seems to depend on the ligand concentration. Irrespective of ligand concentration, compounds **4** (Fig. 2, panels **c** and **e**) and **6** (Fig. 2, panels **d** and **f**) inhibited the androgen-triggered effect on cell cycle markers. Expectedly^25^, expression of Cdk4 was similar under the different experimental conditions (Fig. 2, panels c–f). Quantitative analysis from different Western blots was done and data presented in the Supplementary Information.

Since LNCaP cells express the isoform β of ER, while do not express ERα (Figure S5 in Supplementary Information)^33,40^, we also analyzed the effect of compounds on estradiol-induced DNA synthesis in LNCaP cells. Estrogens induced a significant increase in BrdU incorporation, which was slightly inhibited by the compounds **4** or **6** (Fig. 2g). Both the compounds did not affect the BrdU incorporation in cycling or androgen-stimulated PC3 and DU145 cells (Figure S5 in Supplementary Information). These cells, indeed, do not express AR and are insensitive to androgens, albeit they express similar amounts of TRPM8 (Figure S5 in Supplementary Information). Thus, TRPM8 antagonists impair the mitogenic response and cell cycle progression controlled by AR in LNCaP cells, leaving unaffected the DNA synthesis in AR-negative PC cells.

**Figure 3:**
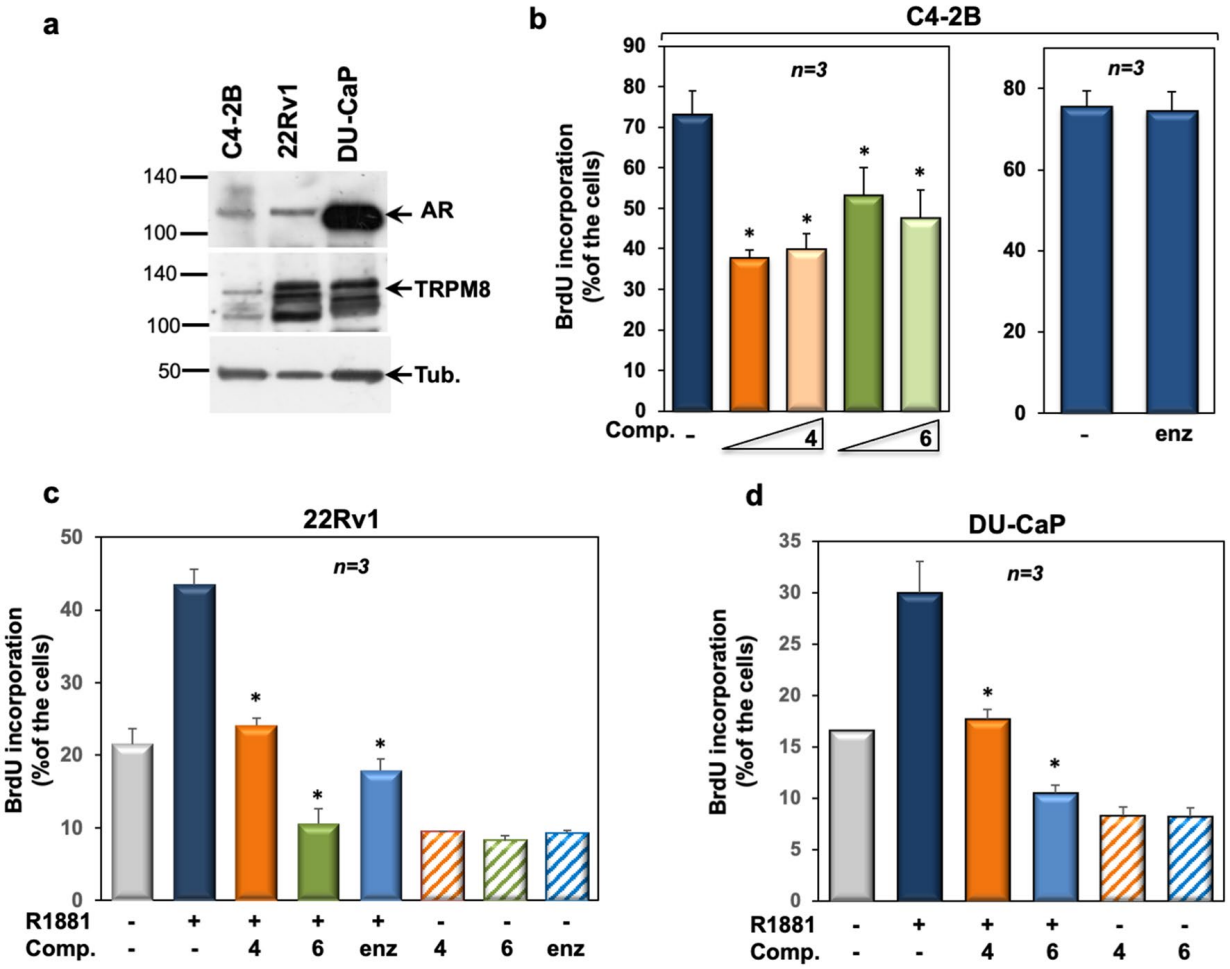
TRPM8 antagonists, 4 and 6, affect the DNA synthesis in various PC-derived cells. In (**a**), lysate protein from C4-2B, 22Rv1 and DU-CaP were analyzed by WB technique, using the antibodies against the indicated proteins. WB are representative of three different experiments and densitometric analysis of data is reported in Supplementary Information. In (**b**), cycling C4-2B cells on coverslips were left un-challenged or challenged for 18 h with increasing (100 nM, 1 μM) concentrations of TRPM8 modulators **4** and **6** (left panel) or enzalutamide (10 μM; right panel). In **c** and **d**, quiescent 22Rv1 (**c**) and DU-CaP (**d**) cells on coverslips were left untreated or treated for 18 h with 10 nM R1881, in the absence or presence of compounds **4** and **6** (at 1 μM) or enzalutamide (at 10 μM). In (**b–d**), cells were pulsed in vivo with 100 μM BrdU. Its incorporation into newly synthesized DNA was analyzed by IF and expressed as % of total cells. Means and standard errors (SEMs) are shown. *n,* represents the number of experiments. *p < 0.05 for the indicated experimental points *vs.* the corresponding untreated control.

We next used normal epithelial prostate PNT-2 cells, which express appreciable levels of AR and TRPM8 (Figure S6 in Supplementary Information). Although these cells are sensitive to androgen stimulation in terms of DNA synthesis, they scantly responded to our compounds (Figure S6 in Supplementary Information), likely because of the differential regulation of TRPM8 activity reported in normal as well as transformed prostate cells^41^. At last, we used our selected compounds in C4-2B, 22Rv1 and DU-CaP cells. C4-2B cells derive from the LNCaP subline, C4-2 that acquires the metastatic potential targeting the bone when subcutaneously or orthotopically inoculated into both castrated and intact athymic male nude mice. In contrast with C4-2 cells that grow in the absence of androgens, but yet respond to manipulation of androgen levels, C4-2B cells mimic the natural course of PC progression from the androgen-dependence to -independence^42^. 22Rv1 cells, instead, derive from a xenograft that was serially propagated in mice after castration-induced regression and relapse of the parental, androgen-dependent CWR22 xenograft. 22Rv1 cells express the AR, release prostate specific antigen (PSA) and weakly proliferate in response to androgens^43^. At last, DU-CaP cells derive from a metastatic lesion to the dura mater of a patient with hormone refractory PC^44^. Albeit at different extent, the employed cell lines express significant levels of AR as well as TRPM8 (Fig. 3a). The compounds **4** and **6** inhibited the BrdU incorporation in cycling C4-2B (left section in Fig. 3b), with a more robust and significant effect exerted by the compound **4**. We did not observe any effect in C4-2B cells treated with enzalutamide (right section in Fig. 3b) or in cells challenged with 10 nM R1881 (not shown), as these cells are androgen-independent. Figure 3 also show that the compounds **4** and **6** significantly inhibited the androgen-stimulated BrdU incorporation of 22Rv1 (**c**) or DU-CaP (**d**) cells. The finding that TRPM8 antagonists inhibit the mitogenesis of C4-2B (still expressing AR) or 22Rv1 (expressing both AR and its AR-V7 variant)^45^ or DU-CaP (over-expressing AR) cells would be useful in clinical management of CRPC patients, as they often exhibit AR overexpression or the AR-V7 variant, which confers resistance to abiraterone acetate and enzalutamide^46^.

### TRPM8 modulators impair the migration and invasion induced by androgens in LNCaP cells

**Figure 4:**
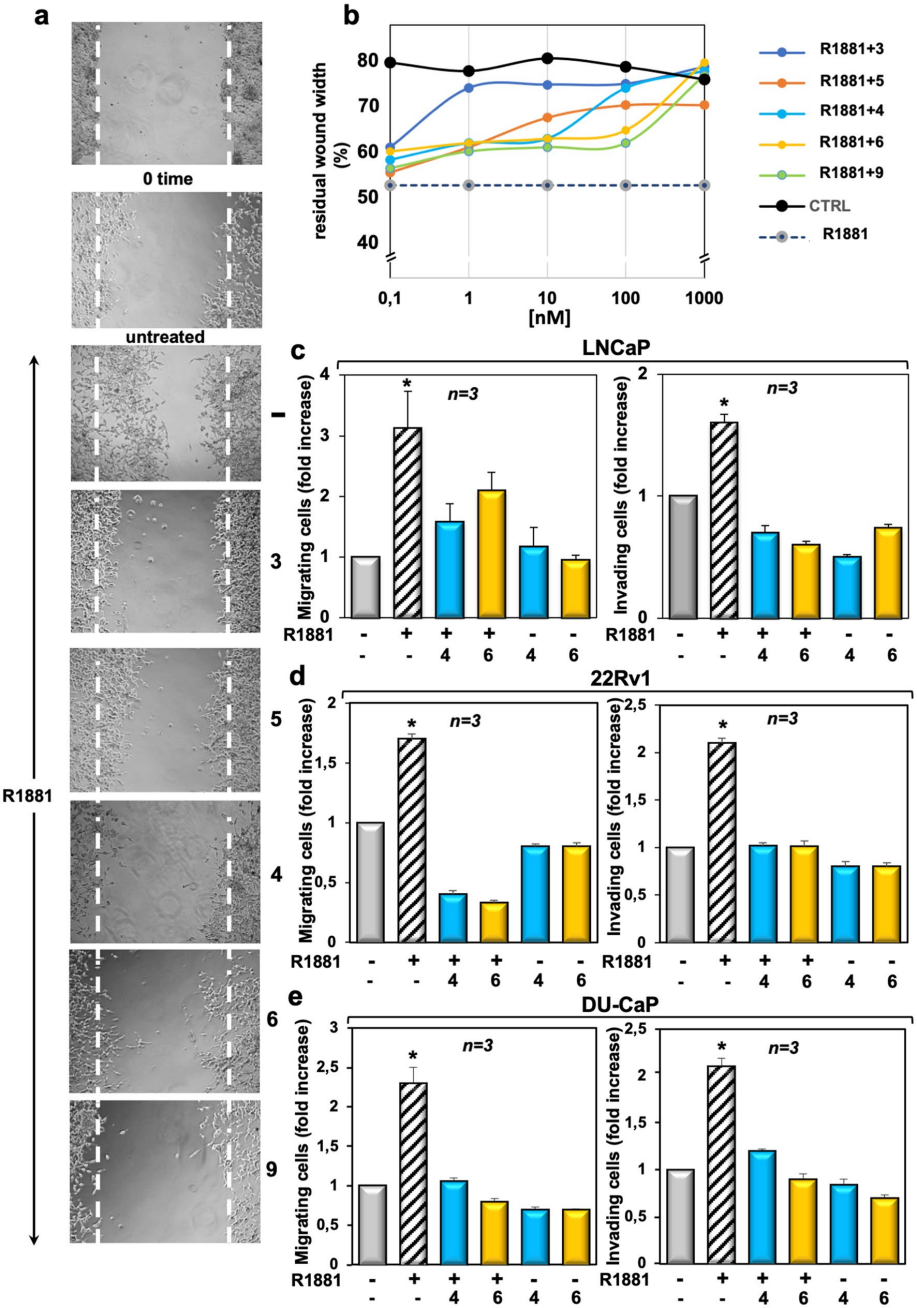
Effect of TRPM8 modulators on migration and invasion induced by androgens in LNCaP cells. Quiescent LNCaP cells were used. In (**a**), cells were wounded and left unstimulated or stimulated with 10 nM R1881 for 20 h, in the absence or presence of the indicated compounds (at 1 μM). In (**b**), wounded cells were left unstimulated or stimulated with 10 nM R1881 for 20 h, in the absence or presence of the indicated concentrations of TRPM8 modulators. In (**a**), phase-contrast images are representative of three different experiments, each in duplicate. In (**b**), the wound area was calculated using Leica Suite Software. Data are presented as % of residual wound width. The standard deviations were < 0,05 for each experimental condition. LNCaP, 22Rv1 and DU-CaP cells were used for migration (left panels in **c**–**e**) or invasion (right panels in **c**–**e**) assays. The cells were left unstimulated or stimulated with 10 nM R1881, in the absence or presence of the indicated compounds (at 1 μM). Migrating or invading cells were detected and counted as reported in “Methods”. Results from three different experiments were collected and expressed as fold increase. Means and SEMs are shown. *n* represents the number of experiments. *p < 0.05 for the indicated experimental points *vs*. the corresponding untreated control.

Androgens increase the motility and invasiveness of various cell types through rapid activation of signaling effectors involved in cell locomotion^27,40^. Therefore, we evaluated the effect of TRPM8 modulators on androgen-induced motility by wound scratch assay in LNCaP cells. A robust increase in motility was observed on hormone challenging of LNCaP cells, as the wound gap was significantly reduced (Fig. 4a). Albeit at different extent, TRPM8 modulators (at 1 μM) inhibited the wound-gap closure in androgen-treated cells (Fig. 4a), with a stronger effect exerted by the compounds **4** and **6**. Quantification of wound scratch assay at different concentrations of TRPM8 modulators was also done and graphically shown (Fig. 4b). The resulting graph confirms that the compounds **4** and **6** exhibited a stronger effect as compared with that obtained by using the other modulators. Irrespective of the experimental conditions, the compounds **4** and **6** did not modify the wound closure in AR-negative PC3 (Figure S7 in Supplementary Information) or DU145 cells (Figure S8 in Supplementary Information).

We next used a Transmigration assay in Boyden chamber. Quiescent LNCaP cells were challenged with 10 nM R1881 and allowed to migrate, in the absence or presence of the indicated compounds (at 1 μM). Results in Fig. 4c (left panel) show that androgens increased by almost 3 folds the number of migrating cells. Compounds **4** and **6** both inhibited the androgen-induced cell migration, leaving unaffected the migration of LNCaP cells when added alone to the cell medium. Again, stimulation of quiescent cells with 10 nM R1881 increased by 1,6-folds the number of invading LNCaP cells in Matrigel. Compounds **4** and **6** reversed such response and showed a negligible effect when used in the absence of hormone (Fig. 4c, right panel). The finding that the compound **6** more robustly inhibits the invasiveness (right panel) than migration (left panel) of LNCaP cells might be due to the stronger effect exerted by this compound on adherent cells. Superimposable results were observed by Transmigration and invasion assays in 22Rv1 (Fig. 4d) or DU-CaP (Fig. 4e) cells. In summary, TRPM8 antagonists interfere in the migratory phenotype stimulated by androgens in PC-derived cells.

### TRPM8 antagonists inhibit the androgen-induced increase of LNCaP cell spheroids size

**Figure 5:**
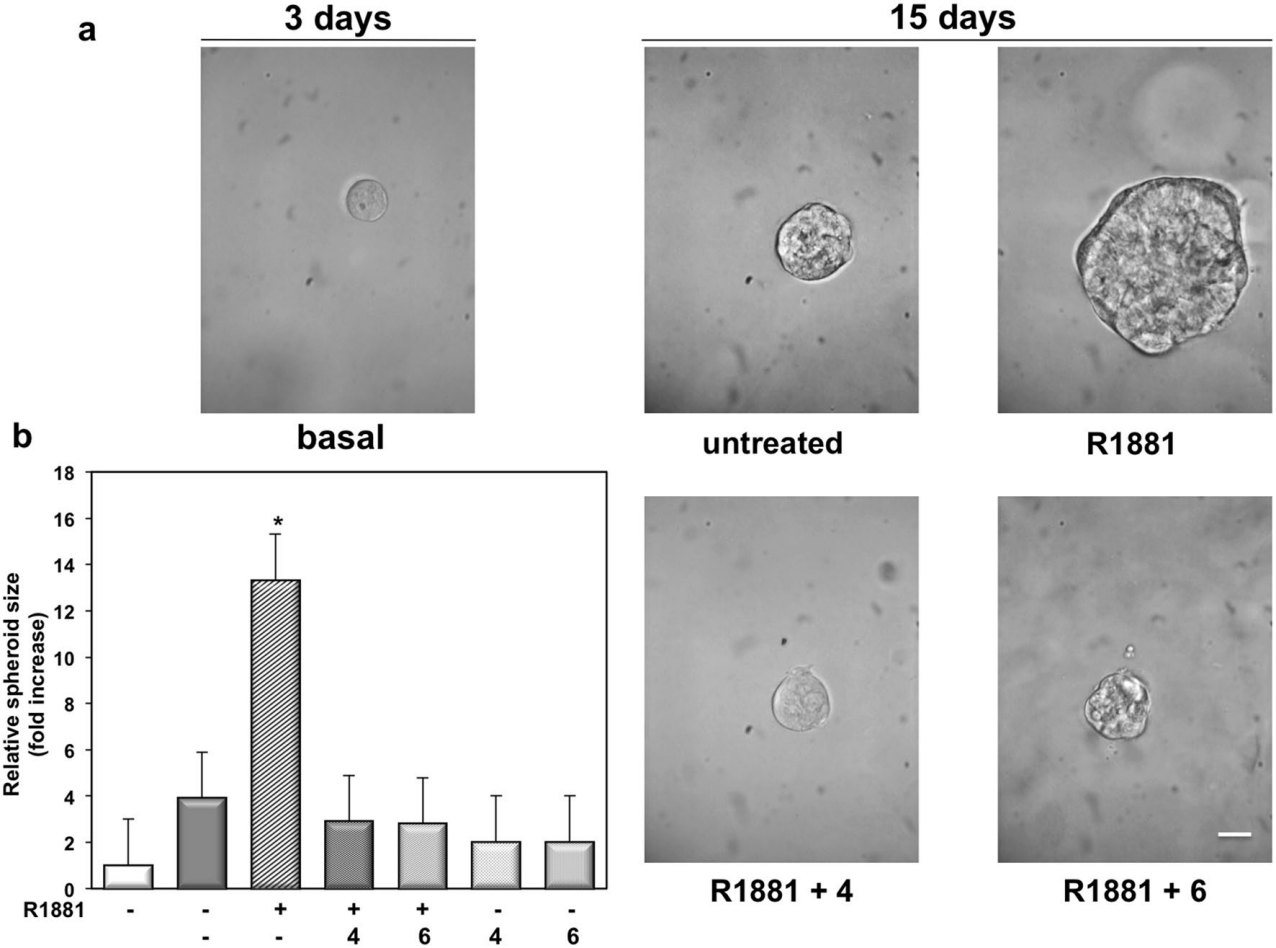
TRPM8 antagonists, 4 and 6, inhibit the androgen-induced increase in LNCaP cell spheroid size. LNCaP cells were employed in 3D cultures and 3 days after the cell’s embedding in Matrigel, representative images were acquired as described in “Methods”. 3D cultures were left untreated or treated with 10 nM R1881, in the absence or presence of the indicated compounds (at 1 μM) for 15 days. Shown are phase-contrast images, representative of 3 different experiments and captured at 15th day. Scale bar, 100 μm. In (**b**), the spheroid size was calculated as described in “Methods” and expressed as fold increase in the relative spheroid size. Means and SEMs from three different experiments are shown. * p < 0.05 for the indicated experimental points *vs*. the corresponding untreated control.

We next generated miniaturized LNCaP cultures in extracellular matrix (ECM) and observed a 3D structure after 3 days culture of LNCaP cells (Fig. 5a). Spheroids were then left untreated or treated with 10 nM R1881, in the absence or presence of 1 μM of the TRPM8 antagonists, **4** or **6**. Changes in dimension and structure of organoids were monitored for additional 15 days. Phase-contrast microscopy (Fig. 5a), together with quantification of data (Fig. 5b), shows that androgen stimulation resulted in a robust increase of the LNCaP cell spheroid size. The compounds **4** and **6** both abolished the androgen-induced effect (Fig. 5a,b), leaving unaffected this response when used in the absence of hormone (Fig. 5b). Consistent with our previous findings^47^, only a weak response on LNCaP spheroid size was detected at 1 nM R1881. The compounds **4** and **6** reverted this effect (Figure S9 in Supplementary Information). In summary, findings here obtained further make the TRPM8 as a putative target in PC.

### TRPM8 antagonists inhibit the rapid androgen action in LNCaP cells

**Figure 6:**
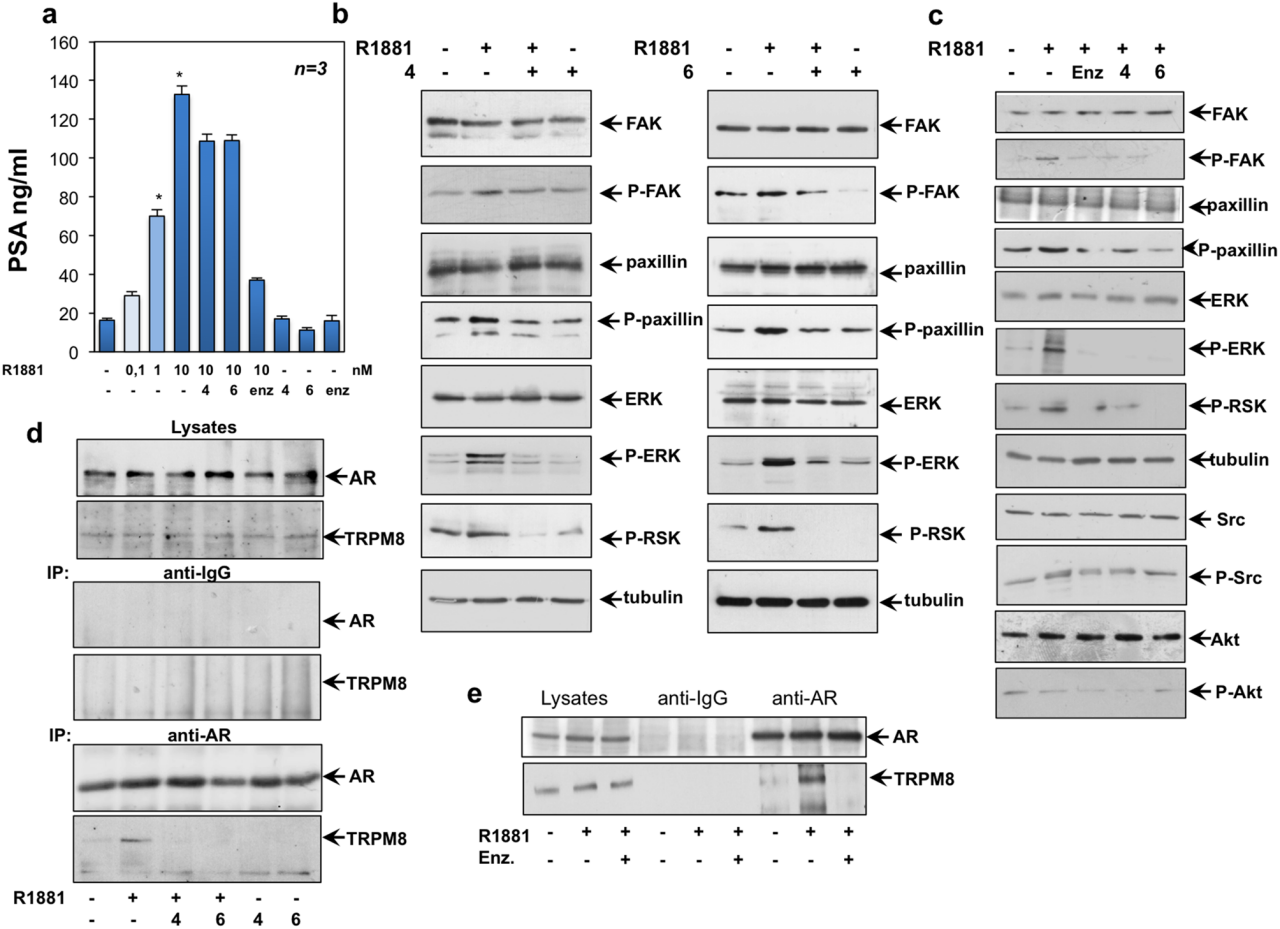
TRPM8 antagonists inhibit the rapid androgen action by disrupting the ligand-induced AR/TRPM8 complex assembly in LNCaP cells. In (**a–e**), quiescent LNCaP were used. When indicated the compounds **4** and **6** were used at 1 μM. Enzalutamide (enz) was added at 10 μM. In (**a**)**,** cells were left untreated or treated with increasing R1881 concentration (0,1, 1 or 10 nM), in the absence or presence of the indicated compounds for 48 h. Conditioned media were collected and PSA was assayed as described in “Methods”. Means and SEMs from three different experiments are shown. *p < 0.05 for the indicated experimental points *versus* the corresponding untreated control. In (**b**–**e**), cells were unstimulated or stimulated with 10 nM R1881, in the absence or presence of the indicated compounds for 10 min. Lysate proteins were analyzed by WB technique using the antibodies against the indicated proteins. P-FAK stands for Tyr 397-P-FAK; P-paxillin stands for Tyr 118-P-paxillin; P-RSK stands for P-Ser 380 RSK; P-ERK stands for phospho-p44/42 MAPK (Erk1/2) (Thr202/Tyr204); P-Src stands for P-Tyr 416 Src and P-Akt stands for P-Ser 473 Akt. The filters were stripped and re-probed using anti tubulin antibody, as loading control. In the upper panel in (**d**) and left section in (**e**), lysate proteins were analyzed by WB using the antibodies against AR or TRPM8. Lysate proteins were immunoprecipitated using the anti-AR antibody (anti-AR, lower panel in (**d**) and right section in (**e**) or control IgG (anti-IgG; middle panel in (**d**) and middle section in **e**). Proteins in immune-complexes were revealed by WB using the antibodies against the indicated proteins. Results in (**b–e**) are representative of 3 different experiments and the densitometric analysis is reported in Supplementary Information.

Androgens modulate the activity of TRPM8 through non-genomic mechanisms^21,48,49^. Therefore, we investigated the mode of action of our compounds. As readout of the androgen-induced gene transcription, we evaluated the effect of compounds **4** and **6** on the androgen-induced PSA secretion from LNCaP cells. By increasing the ligand concentration (from 0.1 to 10 nM R1881), a significant increase in ligand-stimulated gene transcription was detected. At 10 nM ligand concentration, a negligible inhibition of PSA secretion was detected upon addition of the compounds **4** or **6**. By contrast, enzalutamide strongly inhibited the effect exerted by androgens. Neither the two TRPM8 antagonists, nor enzalutamide did modify such response when used alone (Fig. 6a). Thus TRPM8 antagonists do not act on gene transcription mediated by the ligand-bound AR. Because of these results, we next analyzed the effect of compounds **4** and **6** on the androgen-induced rapid actions in LNCaP cells. Albeit at different extent, 10 nM R1881 triggered within 10 min the focal adhesion kinase (FAK) activation as well as paxillin phosphorylation. Both these responses are involved in the hormonal signaling leading to cell motility^50^. Addition of compounds **4** (left section in **b**) or **6** (right section in **b**) impaired the androgen-elicited effect. The compounds did not affect FAK or paxillin phosphorylation when used alone. Using the same lysate proteins, we analyzed the effect of the two compounds on the androgen-triggered extracellular-regulated kinase (ERK) and 90 kDa ribosomal S6 kinase (RSK). Hormone stimulation of LNCaP cells activated within 10 min ERK and RSK, which are needed for the androgen-elicited mitogenesis^28,33^. Treatment of androgen-stimulated cells with the compound **4** (left section in **b**) or **6** (right section in **b**) inhibited both the ERK and RSK activation, leaving unaffected the phosphorylation/activation of the two effectors when used alone. We then compared the effect of TRPM8 antagonists or enzalutamide on the androgen-induced rapid actions in LNCaP cells. Panel **c** in Fig. 6 confirms that hormone stimulation rapidly triggered the activation of FAK, paxillin, ERK, RSK and Src tyrosine kinase. Addition of compounds **4** or **6** or enzalutamide impaired at similar extent the androgen-elicited effect. Hormone stimulation did not affect Akt activation, because of the PI3-K pathway deregulation in LNCaP cells^26^. In sum, while enzalutamide reverses both transcriptional and non-transcriptional events mediated by AR, the antagonists of TRPM8 only interfere in the rapid signaling circuits activated by androgens in target cells, leaving unaffected the AR-mediated gene transcription.

These results raise the question of how the androgen/AR axis intersects TRPM8 in LNCaP cells. To address this issue, we investigated by Co-IP approach the complexation between AR and TRPM8 in LNCaP cells. In a preliminary time-course experiment, we observed that the maximal AR/TRPM8 Co-IP occurred within 10 min androgen stimulation of quiescent LNCaP cells to return to the basal level upon 20 min hormone challenging (not shown). Therefore, we used that time-point to investigate the effect of compounds **4** or **6**. Regardless of experimental conditions, similar amounts of AR or TRPM8 were detected in lysate proteins (upper section in Fig. 6d). Ten minutes androgen stimulation significantly increased the Co-IP of AR with TRPM8. The compounds **4** or **6** abolished this association, while they did not affect the AR/TRPM8 complex assembly when used in the absence of hormone (lower section in Fig. 6d). Similarly, enzalutamide perturbed the androgen-induced AR/TRPM8 complex assembly (Fig. 6e). The absence of AR or TRPM8 in lysate proteins immunoprecipitated with control antibodies confirms the specificity of our approach (middle section in **d** and panel **e**).

### TRPM8 antagonists inhibit the androgen-triggered intracellular calcium increase in LNCaP cells

**Figure 7:**
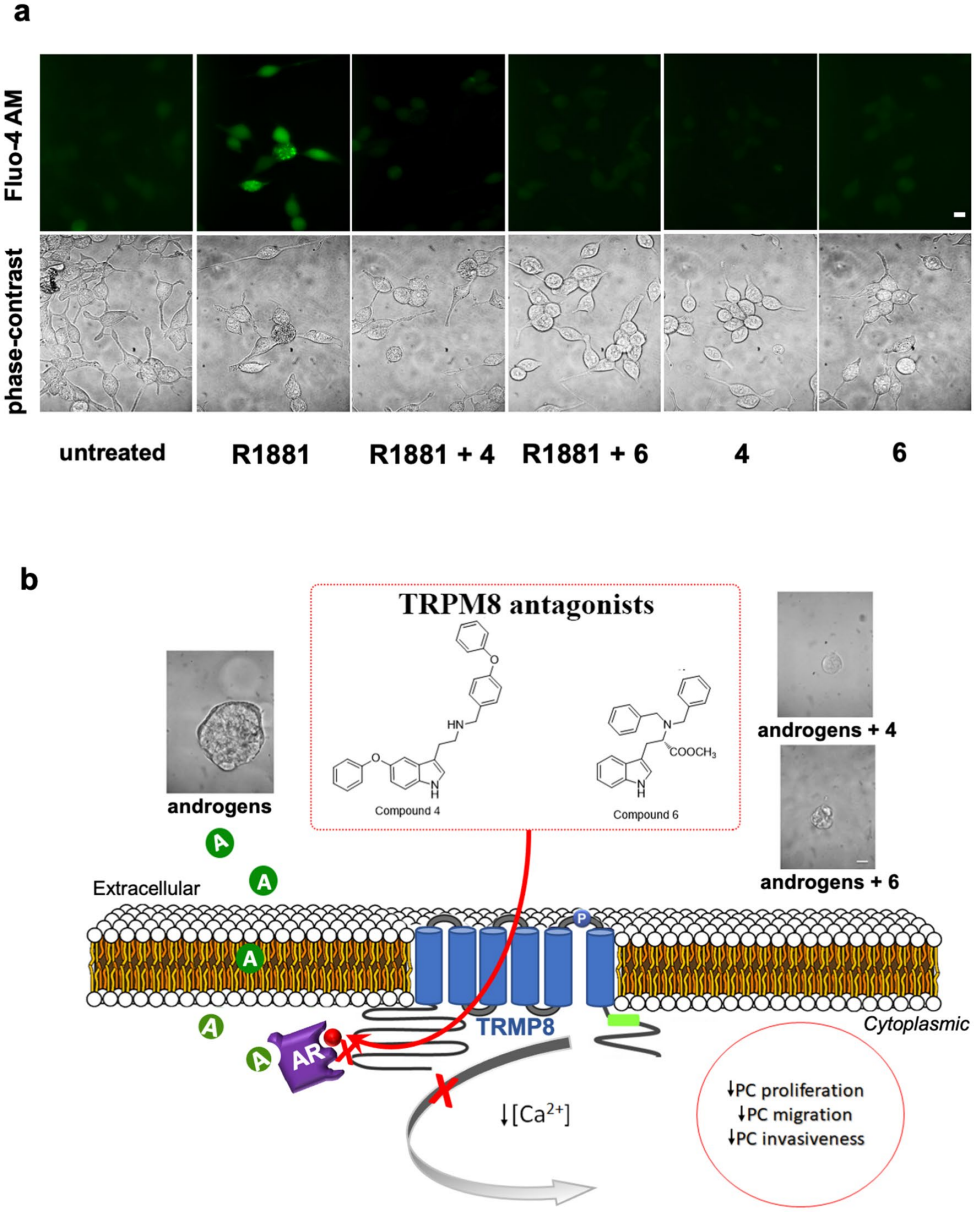
TRPM8 antagonists inhibit the androgen-induced increase of intracellular Ca^2+^ levels in LNCaP cells. In (**a**), LNCaP cells were made quiescent and loaded with 1 µM 4-Fluo AM, as described in “Methods”. Cells were left untreated or treated for 240 s with 10 nM R1881, in the absence or presence of the indicated compounds (at 1 μM). Different fields were analyzed by fluorescence (upper images) or phase-contrast (lower images) microscopy, as described in “Methods”. Representative images were acquired using a DFC 450C camera (Leica). Bar, 10 μM. In (**b**), the mechanism of TRPM8 antagonist action in PC cells is shown. Androgens rapidly induce association of AR with TRPM8 in androgen-sensitive PC cells. Once assembled, this complex leads to intracellular calcium increase and promotes proliferation as well as pro-metastatic phenotype of PC cells. By perturbing the androgen-challenged AR/TRPM8 complex assembly, TRPM8 antagonists (compounds **4** and **6**) lower the intracellular calcium levels and impair the proliferation, invasion and growth in 3D of PC cells.

We finally evaluated the impact of androgen-triggered AR/TRPM8 complex assembly on intracellular calcium levels. Imaging experiments using the Ca^2+^ indicator Fluo 4-AM were then done. Fluorescence images (Fig. 7a) show that 10 nM R1881 increased within 240 s the intracellular Ca^2+^ levels, as compared with the untreated, quiescent LNCaP cells. Compounds **4** or **6** reduced the androgen effect, while any response was detected by addition of TRPM8 antagonists to quiescent LNCaP cells. In summary, we posit that the androgen-triggered AR/TRPM8 complex assembly modulates the calcium channels, thereby inducing an increase in intracellular calcium levels. Interference in the androgen-activated AR/TRPM8 complex by TRPM8 antagonists reverses this effect (Fig. 7b).

## Discussion

The role of TRPM8 in PC is not completely understood^9,15^. In this report, we aim at analysing the intersection of TRPM8 with androgen/AR axis in PC cells. Therefore, we used several PC-derived cell lines, representative of both androgen-dependent (LNCaP, 22Rv1 and DU-CaP cells) as well as the castrate-resistant C4-2B cells that are androgen-independent, albeit they still express AR. At last, PC3 and DU145 cells were used, since they do not express AR and are insensitive to hormone stimulation. Normal prostate PNT-2 epithelial cells were also employed. All the cell lines express appreciable amounts of TRPM8, as assessed by WB analysis of lysate proteins. Notably, LNCaP, 22Rv1, DU-CaP and C4-2B cells express AR and are almost all sensitive to androgens, except for C4-2B cells. In these cells, we have analyzed the biological effects of new synthesized compounds, which modulate the Ca^2+^ permeable, non-selective cation TRPM8 channels. The presented findings show that nearly all the synthesized modulators inhibit the DNA synthesis elicited by androgens in LNCaP cells. Because of the higher potency and efficacy of compounds **4** and **6**, we have used these compounds as the most suitable modulators throughout our experiments.

PC often exhibits cyclin D over-expression or lose cell-cycle negative regulators, such as p21 and p27^51^. The compounds **4** and **6** significantly impair the androgen-induced up-regulation of cyclin D1 as well as p27 down-regulation in LNCaP cells. The lead compounds specifically interfere with the androgen-induced mitogenic effects, leaving unaffected the DNA synthesis elicited by estrogens in LNCaP cells, likely because other channels, such as TRPM6^52^ and TRPM4^53^ are involved in the estrogen signaling. Of note, the compounds **4** and **6** do not affect the S-phase entry of AR-negative PC3 and DU145 cells, while they significantly inhibit the mitogenesis of cycling C4-2B cells or androgen-stimulated 22Rv1 and DU-CaP cells. These latter findings are of interest in therapeutic approach of PC, which often develops resistance to the androgen deprivation therapy (ADT), although still expressing AR. In other cases, instead, PC might exhibit ADT resistance, because of the AR-V7 variant expression. As before described (see the Result’s section), C4-2B and 22Rv1 cells are representative of the two different clinical patterns.

Ten nM testosterone positively affects the TRPM8-mediated migration in PC cells^21^. Consistent with these findings, our selected compounds impair the motility and invasiveness induced by 10 nM R1881 in AR-positive PC cells, but not AR-negative PC3 or DU145 cells. At last, a strong inhibitory effect is observed in 3D model, as the compounds reverse the androgen-induced increase in LNCaP cell spheroid size, further supporting the applicative potential of **4** and **6** compounds.

Steroid hormones regulate the Transient Receptor Potential (TRP) channels through rapid, non-transcriptional mechanism. Nevertheless, few reports have so far investigated the non-transcriptional effects related to the TRPM subfamily of TRP proteins. As before discussed, TRPM4 and TRPM6 have been linked to non-transcriptional estrogen action in different cell types and different experimental settings. Pregnenolone sulfate, the mother of all steroid hormones, rapidly and reversibly activates TRPM3, a divalent-permeable cation channel, thus inducing a rapid calcium influx as well as insulin secretion from pancreatic islets^54,55^. These findings identified TRPM3 as the playmaker between steroidal and insulin endocrine systems. A role for pregnenolone sulfate in activation of TRPM3 has been recently confirmed in somatosensory afferent neurons^56^. Relevant to our data, 10 nM testosterone challenges the interaction between AR and TRPM8 within lipid rafts microdomains of PC3 cells engineered to overexpress AR^21^. We here dissected an endogenous non-genomic mechanism of the TRPM8 channel regulation by androgens in PC cells. Ten nanomolar R1881 induces within minutes a significant increase in AR/TRPM8 Co-Ip in LNCaP cells. Once assembled, the complex triggers activation of various downstream effectors involved in the androgen-signaling leading to proliferation and motility of target cells. These findings encourage the development and use of novel TRPM8 antagonists, which perturb the androgen-elicited rapid responses, without affecting the secretion of PSA and gene transcription in PC cells.

At last, to assess the effect of androgens on cytosolic Ca^2+^ mobilization caused by TRPM8 activation we have also conducted Ca^2+^ imaging experiments in LNCaP cells. Hormone stimulation of PC cells a rapid elevation of cytosolic Ca^2+^ concentration in a pattern that depends on AR/TRPM8 complexation, since the TRPM8 antagonists, which perturb the complex assembly, reverse such increase. These results make TRPM8 as the molecular link between androgen and calcium systems. Noteworthy, the Ca^2+^ signaling intersects sex steroid receptor-mediated events^57–59^ and controls the behavior of cancer cells^60^. Thus, by altering the expression and/or activity of Ca^2+^ channels, our selected antagonists affect Ca^2+^ homeostasis with important consequences in cell outcome. These findings, together with the observation that TRPM8 sensitizes therapy-refractory models of PC to radio-, chemo- or hormonal treatments^15^, make TRPM8 as a ‘druggable’ candidate in PC. Therefore, the discovery of new selective TRPM8 antagonists represents a promising approach in PC patient’s management. Moreover, our compounds combine the selective modulation of non-genomic actions mediated by AR with the targeted release of intracellular calcium. This bifunctional approach may be more effective than the currently used ADT. At last, the present results together with the identification of TRPM8 mRNA as a bloodstream signature for high-risk PC patients^61^, pave the way for new options in patient’s stratification and treatment guidance.

## Methods

### Chemicals and reagents

Unless otherwise stated, reagents, starting materials, and solvents were from Sigma-Aldrich (St. Louis, MO, USA). Reactions were carried out with magnetic stirring in round-bottomed flasks. Analytical thin layer chromatography (TLC) was performed on pre-coated glass silica gel plates 60 (F254, 0.25 mm, VWR International). Purifications were performed by flash column chromatography on silica gel (230−400 mesh, Merck Millipore). 1D and 2D NMR spectra were recorded with Bruker Avance (400 MHz) spectrometer, at room temperature. Chemical shifts are reported in δ values (ppm) relative to internal Me_4_Si, and J values are reported in hertz (Hz). The following abbreviations are used to describe peaks: s (singlet), d (doublet), dd (double doublet), t (triplet), q (quartet), and m (multiplet). HR-MS experiments were performed using an LTQ-Orbitrap-XL-ETD mass spectrometer (Thermo Scientific, Bremen, Germany), using electrospray ionization. Analytical RP-HPLC was performed on a Phenomenex Synergi Fusion RP-80A (75 mm × 4.6 mm, 4 μm), with a flow rate of 1 mL/min, using a tunable UV detector at 254 nm. Mixtures of CH_3_CN and 0.05% TFA in H2O were used as mobile phase. All compounds showed a purity ≥ 95%. The synthetic androgen R1881 was used at 1 or 10 nM. Enzalutamide (Selleckchem, Munich, Germany) was used at 10 μM.

### Cell cultures

LNCaP and Cos-7 cells were from Cell Bank Interlab Cell Line Collection (ICLC- Genova—Italy). 22Rv1 and C4-2B cells were from LGC Standards S.r.L. DU-CaP cells^62^ were obtained from Prof J. A. Schalken. PNT-2 cells were from Sigma Aldrich. Cells were authenticated by the Supplier through DNA profiling by short tandem repeats (STRs) and maintained at 37 °C in humidified 5% CO_2_ atmosphere. Media and supplements used for these cells were from Gibco (Thermofisher; Waltham, MA USA). LNCaP, C4-2B, 22Rv1, DU-CaP and PNT-2 cells were cultured in RPMI-1640 supplemented with 10% foetal bovine serum (FBS), penicillin (100 U/mL), streptomycin (100 U/mL), glutamine (2 mM), sodium pyruvate (1 mM) and non-essential amino acids (0.1 mM). Three days before stimulation, LNCaP, 22Rv1 and DU-CaP cells were made quiescent using phenol red-free RPMI-1640 medium containing 10% charcoal-stripped serum (CSS), penicillin (100 U/mL), streptomycin (100 U/mL) and 0,02 U/L insulin. Twenty-four hours before stimulation, PNT-2 cells were made quiescent using phenol red-free RPMI-1640 medium containing 0,1% charcoal-stripped serum (CSS), penicillin (100 U/mL), streptomycin (100 U/mL) and 0,02 U/L insulin. DU145 cells were cultured in phenol-red DMEM supplemented with 10% FBS, penicillin (100 U/mL), streptomycin (100 U/mL) and glutamine (2 mM). PC3 cells were cultured in RPMI with 10% FBS, penicillin (100 U/mL), streptomycin (100 U/mL) and glutamine (2 mM). Twenty-four hours before stimulation, DU145 cells were made quiescent using phenol red-free DMEM containing penicillin (100 U/mL) and streptomycin (100 U/mL). Forty-eight hours before stimulation, PC3 cells were made quiescent using phenol red-free RPMI-1640 medium supplemented with penicillin (100 U/mL), streptomycin (100 U/mL), and 0,5% charcoal-stripped serum (CSS). The quiescence of PC-derived cells and normal prostate cells was monitored by DNA synthesis analysis. We always used cells that weakly incorporated bromodeoxyuridine (BrdU) under basal conditions (~ 10–20% of total cells). Cos-7 cells were cultured in DMEM supplemented with 10% FBS, penicillin (100 U/mL), streptomycin (100 U/mL), glutamine (2 mM), hydrocortisone (3,75 ng/ml) and insulin (6 ng/ml). In electrophysiology experiments, HEK-293/TRPM8 exon1 K3 cells were used. Cells were cultured in minimum essential medium with Earle’s salts, without L-glutamine (Euroclone) supplemented with 5 mL of 200 mM Ultraglutamine 1 in 0.85% NaCl solution (Lonza), 5 mL of 100 × penicillin/streptomycin (Lonza), 0.2 mL of 10 mg/mL puromycin (InvivoGen; final concentration 0.4 μg/mL), and 50 mL of fetal bovine serum (Sigma-Aldrich, Milan, Italy), as reported^31^. Assessment of channel subtype selectivity was performed by fluorometric experiments using HEK-293 cells lines stably transfected with either hTRPA1 or hNav1.7 and CHO-K1 stably transfected with hTRPV1. HEK-293 cells were cultured in EMEM (MEM Eagle Earl’s salts balanced salt solution, Lonza, Walkersville, USA) containing 5 mL of 200 mM Ultraglutamine1 (Lonza), 5 mL of 100 × penicillin/streptomycin (Lonza), 50 mL of FBS (Euroclone, Milan, Italy), 2 mL of 100 mg/mL G418 (InvivoGen, San Diego, USA), as described^31^. CHO-K1 cells were grown in DMEM F-12 (1:1) mixture (Lonza) containing 5 mL of 100 mM sodium pyruvate (Lonza), 25 mL of 7.5% sodium bicarbonate (Lonza), 6.5 mL of 1 M HEPES (Lonza), 5 mL of 100 × penicillin/streptomycin (Lonza), 50 mL of FBS (Euroclone), 0.25 mL of 10 mg/mL puromycin (InvivoGen), and 0.5 mL of 100 mg/mL zeocin (InvivoGen). L-Menthol (Sigma-Aldrich) was the selected reference agonist. All the cell lines were routinely monitored for Mycoplasma contamination.

### Selectivity assays

Selectivity assays were performed in 384-well clear bottom black walled polystyrene plates, (Thermo Scientific, Waltham, USA) for CHO-K1 cells and in 384-well clear bottom black polystyrene walled poly-D-Lys coated plates (TwinHelix, Rho, Italy) for HEK-293 cells. Consistent with previously described protocols^31^, the compounds were diluted in 96-well U bottom plates (Thermo Scientific), and then transferred into 384-well V bottom polypropylene barcoded plates (Thermo Scientific). The activity of the selected compound over TRPA1 and TRPV1 was assessed by seeding cells in 384 MTP in complete medium (25 μL/well) at a concentration of 10 000 cells/well. The day after, culture medium was removed and cells were loaded with 20 μL/well of 0.5 × calcium sensitive dye (Fluo-8 NW, AAT Bioquest, Sunnyvale, USA), dissolved in the assay buffer. For the assessment of compounds activity over Nav1.7, indeed, cells were seeded at 15 000 cells/well in 384 MTP in complete medium (25 μL/well). One day after seeding, the culture medium was removed and cells were loaded with 20 μL/well of 0.5 × membrane potential dye (FLIPR membrane potential assay kits Blue, Molecular Devices LLC, San Jose, USA) dissolved in assay buffer. Plates were then incubated for 60 min at room temperature in the absence of light. Thereafter, 10 μL/well of test compounds and controls were injected at 3 × concentrations and the emitting fluorescence signal was recorded using FLIPRTETRA apparatus (ForteBio, Fremont, USA). A second injection of 15 μL/well of 3 × reference activator (at ∼EC80) was done by analyzing the emitting fluorescence signal. Selected reference agonists were allyl isothiocyanate (AITC, Sigma-Aldrich), capsaicine (Sigma-Aldrich), and veratridine (Sigma-Aldrich) for TRPA1, TRPV1, and Nav1.7 channels, respectively. Selected reference antagonists for TRPA1, TRPV1, and Nav1.7 channels were, instead, HC-030031 (Sigma-Aldrich), capasazepine (Sigma-Aldrich), and tetrodotoxine (Tocris Bioscience, Bristol, U.K.), respectively. Selectivity of final compounds **3** and **6** has been previously determined^29,30^.

### Patch-clamp experiments

Patch clamp experiments for compounds 4, 5 and 9 were done as described^31^. HEK-293/TRPM8 exon 1 cells were seeded 3 or 4 days before experiment at a concentration of 4 and 2.5 million cells, respectively, onto a T225 flask. Before each experiment, cells were washed two times with D-PBS without Ca^2+^/Mg^2+^ (Euroclone, Milan, Italy) and trypsinized with trypsin−EDTA (Sigma-Aldrich; diluted 1/10). Cells were suspended in 25 mL of EX-CELL ACF CHO medium (Sigma-Aldrich, Milan, Italy), 0.625 mL of HEPES (Lonza, Walkersville, USA), 0.25 mL of 100 × penicillin/streptomycin (Lonza, Walkersville, USA), 0.1 mL of soybean trypsin inhibitor 10 mg/mL (Sigma-Aldrich), and placed on the automated patch-clamp platform (QPatch 16X, Sophion Bioscience, Ballerup, Denmark). A stock solution of L-Menthol (1 M, 100% DMSO), used as reference agonist, was freshly prepared. A diluted stock of 300 mM was prepared from the 1 M stock in 100% DMSO, and a final dilution was obtained using the extracellular solution to give a working concentration of 300 μM (1:1000, 0.1% final DMSO concentration). Stock solutions of the testing compounds (10 mM; 100% DMSO; stored at − 20 °C) were freshly prepared, as well. Further dilution of these stock solutions to a final concentration of 300 μM was performed from the 10 mM stock in 100% DMSO, and the working dilutions were performed just before the experiments using the extracellular solution containing 300 μM menthol. The highest concentration tested was 300 nM, with serial dilutions (1:10). DMSO was balanced to keep it constant throughout all the solutions in the same experiment (0.2% final DMSO concentration). Standard whole-cell voltage clamp experiments were performed at room temperature using the multihole technology. For the voltage clamp experiments on human TRPM8, data were sampled at 2 kHz. After establishment of the seal and the passage in the whole cell configuration, the cells were subjected to a voltage ramp (20 ms step at − 60 mV; 100 ms ramp − 60/ + 100 mV; 20 ms step at + 100 mV; return to − 60 mV) every 4 s. The antagonistic effect on human TRPM8 current of target compounds was evaluated after application of the agonist (menthol, 300 μM), in the absence or presence of increasing concentrations of the compound under investigation. Outward current evoked by the voltage ramp, measured in the step at + 100 mV were measured. The intracellular solution contained (mM) 135 CsCl, 10 BAPTA, 10 HEPES, 4 Na_2_ATP (pH 7.2 with CsOH). The extracellular solution contained (mM) 145 NaCl, 4 KCl, 1 MgCl_2_, 2 CaCl_2_, 10 HEPES, 10 glucose (pH 7.4 with NaOH). TRPM8 modulation by final compounds **3** and **6** has been previously determined by patch clamp measurements^29,30^.

### Wound scratch, migration and invasion assays

In wound scratch analysis, 1.8 × 10^5^ cells were seeded in a 24-well plate and made quiescent. Cells were then wounded using 10 µl sterile pipette tips and washed with PBS. They were left un-stimulated or stimulated for the indicated times, in the absence or presence of the selected compounds. To avoid cell proliferation, cytosine arabinoside (Sigma-Aldrich) at 50 μM (final concentration) was included in the cell medium. Different fields were analysed by contrast-phase microscopy, as below described in this section. Migration and invasion assays were done as reported^27^. In migration assay, we used 3 × 10^4^ cells in collagen pre-coated Boyden’s chambers with 8 μm polycarbonate membrane (Falcon). The indicated stimuli were added to the upper and the lower chambers. Cytosine arabinoside (at 50 μM) was included in the cell medium. After 7 h, non-migrating cells from the membrane upper surface were removed using a sterile cotton swab. In invasion assay, we used 5 × 10^4^ cells in Boyden’s chambers with 8 μm polycarbonate membrane (Falcon) pre-coated with growth factor reduced- and phenol red-free Matrigel (Corning). The indicated stimuli were added to the upper and the lower chambers. Cytosine arabinoside (at 50 μM) was included in the cell medium. After 24 h, non-invading cells from the membrane upper surface were removed using a sterile cotton swab. In migration and invasion assays, the membranes were fixed for 20 min in 4% paraformaldehyde, stained with Hoechst (Sigma-Aldrich), removed with forceps from the companion plate and mounted. Migrating and invading cells were scored by fluorescence microscopy, as below described in this section.

### 3D culture in ECM

LNCaP cell spheroids were generated as reported^26^. Briefly, 2 × 10^4^ LNCaP cells were mixed with 200 μL of phenol-red free growth factor-reduced Matrigel and 50 μL of organoid plating medium in each well. The mixture was pipetted in 24-well plate and allowed to solidify for 45 min at 37 °C, before the addition of 400 μL organoid plating-medium, which was made using phenol red-free DMEM/F12 medium, containing 7% CSS, penicillin (100 U/mL), streptomycin (100 U/mL), diluted GlutaMAX 100X (Gibco), 10 mM Hepes, B27 supplement (50 × stock solution; Thermofisher, Waltham, Massachusetts, USA), 1 M nicotinamide (Sigma-Aldrich), 500 mM N-acetylcysteine (Sigma-Aldrich) and 10 μM Y-27632 (Millipore, Burlington, MA, USA). After 3 days, the organoid-plating medium was replaced with a similar medium in the absence of N-acetylcysteine and Y-27632. At the 3rd day, spheroids/organoids were untreated or treated with the indicated compounds. The medium was changed every 3 days and fields were analyzed by phase-contrast microscopy.

### Phase-contrast microscopy

In wound scratch assay, different fields were analysed using DMIRB inverted microscope (Leica) equipped with N-Plan 10 × objective (Leica), as reported^49^. LNCaP organoids were analysed using the same microscope equipped with C-Plan 40 × or HCX PL Fluotar 63 × objectives (Leica). Images were captured using a DFC 450C camera (Leica) and acquired using the Application Suite Software (Leica). They are representative of three different experiments. The relative organoid size was calculated using the same software and expressed as a fold increase over the basal organoid size (measured at 3th day). The wound gap was calculated using Image J Software and expressed as % of the decrease in the wound area. Migrating or invading cells from at least 30 fields/each membrane were scored, using a DMLB (Leica) fluorescent microscope, equipped with HCPL Fluotar 20 × objective.

### Fluorescent Ca^2+^ imaging

LNCaP cells in 6 multi-wells were made quiescent and incubated for 60 min at 37 °C with 1 µM 4-Fluo AM (Abcam, ab241082). Cells were washed twice with Hank’s Balanced salt solution (HBSS) to remove any dye non-specifically associated with the cell surface. They were incubated with medium for 30 min to allow complete de-esterification of intracellular AM esters, and then left untreated or treated for 240 s with 10 nM R1881, in the absence or presence of the indicated compounds. Different fields were analyzed using DMIRB Leica (Leica) microscope equipped with C-Plan × 40 or HCX PL Fluotar × 63 objectives (Leica). IF microscopy images were generated using a DFC 450C camera (Leica).

### DNA synthesis and immunofluorescence (IF)

Cells on coverslips were pulsed in vivo with BrdU (Sigma-Aldrich; at 100 μM, final concentration) and left unchallenged or challenged with 10 nM R1881 for 18 h, in the absence or presence of the indicated compounds. Cells were fixed, permeabilized, washed and the BrdU incorporation was analysed as reported^63^. Nuclei were stained with Hoechst and cells on coverslips were scored by IF microscopy, using a DMLB Leica (Leica) fluorescent microscope, equipped with HCX PL Apo 63 × oil objective. Different fields from each coverslip were analyzed and BrdU incorporation was calculated by the formula: percentage of BrdU-positive cells = (No. of BrdU-positive cells/No. of total cells) × 100. Data in the graphs derive from at least 1000 scored cells for each coverslip. Representative images were captured using DC480 camera (Leica) and acquired using Leica Suite software. They are representative of three different experiments.

### Prostate specific antigen (PSA) assay

It was done in culture medium using the PSA ELISA kit (Abnova-Taiwan Corporation, Taiwan), according to the manufacturer’s instructions. Released PSA was analyzed at 450 nm using the EnSpire Multimode Plate Reader (Perkin-Elmer, U.S.A.).

### Lysates, immune-precipitation (IP), co-immune-precipitation (Co-IP) and Western blot (WB)

All performed as reported^40^. The mouse monoclonal anti-AR (441; Santa Cruz) antibody was used in Co-Ip or WB approaches. TRPM8 was detected using the rabbit polyclonal anti-TRPM8 (NBP1-97311; Novus Biological). ERK phosphorylation was revealed using the mouse monoclonal anti p-ERK (sc-7383; Santa Cruz) antibody. The rabbit polyclonal anti-ERK (C-14; Santa Cruz), antibody was used to detect total ERK. FAK and P-Tyr397 FAK were detected using the mouse monoclonal anti-FAK (Clone 77/610077 BD Transduction Laboratories) or anti P-Tyr397 FAK (Clone 14/611722; BD Transduction Laboratories) antibodies, respectively. P-Tyr118 paxillin and total paxillin were detected using the rabbit polyclonal anti-P-Y118 (#2541, Cell Signaling) or the mouse monoclonal anti-paxillin (clone 349; BD Biosciences) antibodies, respectively. P-Ser 380 RSK, P-Ser 473 Akt and Akt were revealed using the rabbit polyclonal anti P-Ser380 RSK (#9341; Cell Signaling), anti P-Ser 473 Akt (#9271S; cell signaling) and anti Akt (#9272; Cell Signaling) antibodies, respectively. P-Tyr 416 Src and Src were detected using the rabbit monoclonal anti P-Tyr 416 Src (#6943; Cell Signaling) or the mouse monoclonal anti Src (Calbiochem) antibodies, respectively. ERα and ERβ were detected using the rabbit polyclonal anti-ERα (543; Santa Cruz) or anti-ERβ (Upstate Biotechnology, U.S.A.) antibodies. Cyclin D1, p27 and Cdk4 were stained using the mouse monoclonal anti-cyclin D1 (clone AM29; from Zymed Laboratories Inc.), the rabbit polyclonal anti-p27 (C-19; Santa Cruz) or anti Cdk-4 (c-22; Santa Cruz) antibodies, respectively. The mouse monoclonal anti-tubulin (Sigma-Aldrich) antibody was used to detect tubulin. The ECL system (GE Healthcare) was employed to reveal immune-reactive proteins. Densitometric analysis of WB was performed by using Image J Software (https://imagej.nih.gov/ij).

### Statistical analysis

Experiments were performed in triplicate. Resulting data are presented as the mean ± standard deviation. The differences between values observed after the various treatments were analyzed using the paired Student's *t* test. *p* value < 0.05 was considered significant.

## Supplementary Information

Supplementary Information.

## Data Availability

The datasets used and/or analyzed during the current study are included within the article and its additional file. They are available from the corresponding Authors on reasonable request.

## Abbreviations

AR: Androgen receptor
ARE: Androgen responsive element
ECM: Extracellular matrix
ER: Estradiol receptor
ERK: Extracellular-regulated kinase
FAK: Focal adhesion kinase
IF: Immunofluorescence
PC: Prostate cancer
RSK: 90KDa ribosomal S6 kinase
TRP: Transient receptor potential
TRPM8: Transient receptor potential melastatin-8
WB: Western blot
