# Supplementary material for: Therapeutic potential of TRPM8 antagonists in prostate cancer
Source: Sci Rep. 2021 Dec 1;11:23232. doi: 10.1038/s41598-021-02675-4 (PMC8636514; doi:10.1038/s41598-021-02675-4)
Supplement: Supplementary file 1 — Supplementary Information. [file 41598_2021_2675_MOESM1_ESM.pdf]

THERAPEUTIC POTENTIAL OF TRPM8 ANTAGONISTS IN PROSTATE CANCER.

Marzia Di Donato[1°], Carmine Ostacolo[2°], Pia Giovannelli [1], Veronica Di Sarno[3,] Isabel M. Gomez Monterrey[2], Pietro Campiglia[3,] Antimo Migliaccio[1], Alessia Bertamino[3§] and Gabriella Castoria[1§]

[1] Department of Precision Medicine, School of Medicine- University of Campania 'L. Vanvitelli', Via L. De Crecchio 7, 80138 Naples

[2]Department of Pharmacy, University Federico II of Naples, Via D. Montesano 49, 80131, Naples

[3]Department of Pharmacy, University of Salerno, Via G.Paolo II, 84084, Fisciano (SA)

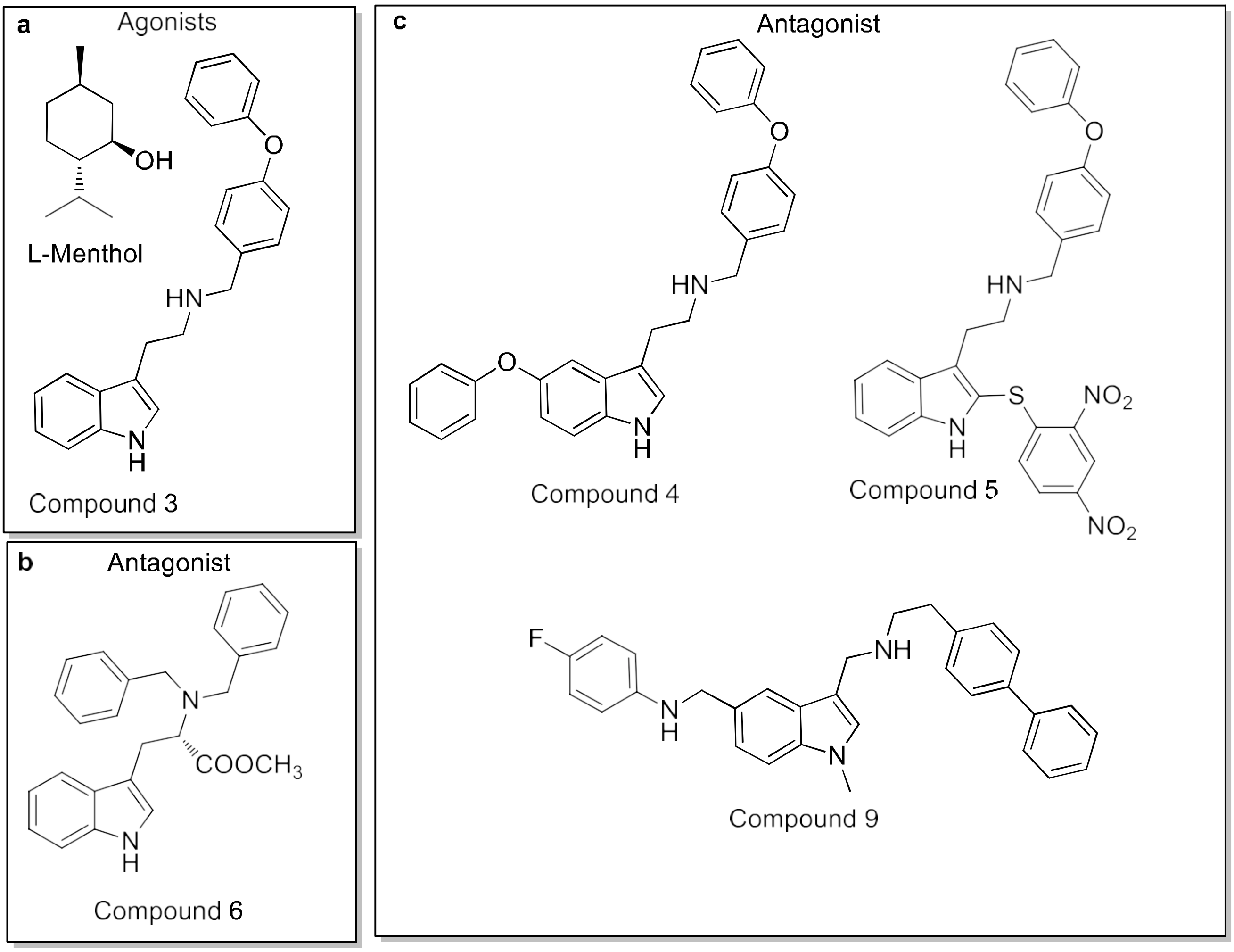

Figure S1: Molecular structures of the tested compounds. Panel **a**: previously described TRPM8 agonists; Panel **b**: previously described TRPM8 antagonist. Panel **c**: newly characterized TRPM8 antagonists.

Chemistry

Figure S2

Synthesis of the compounds was accomplished following the general procedure described in Figure S2.

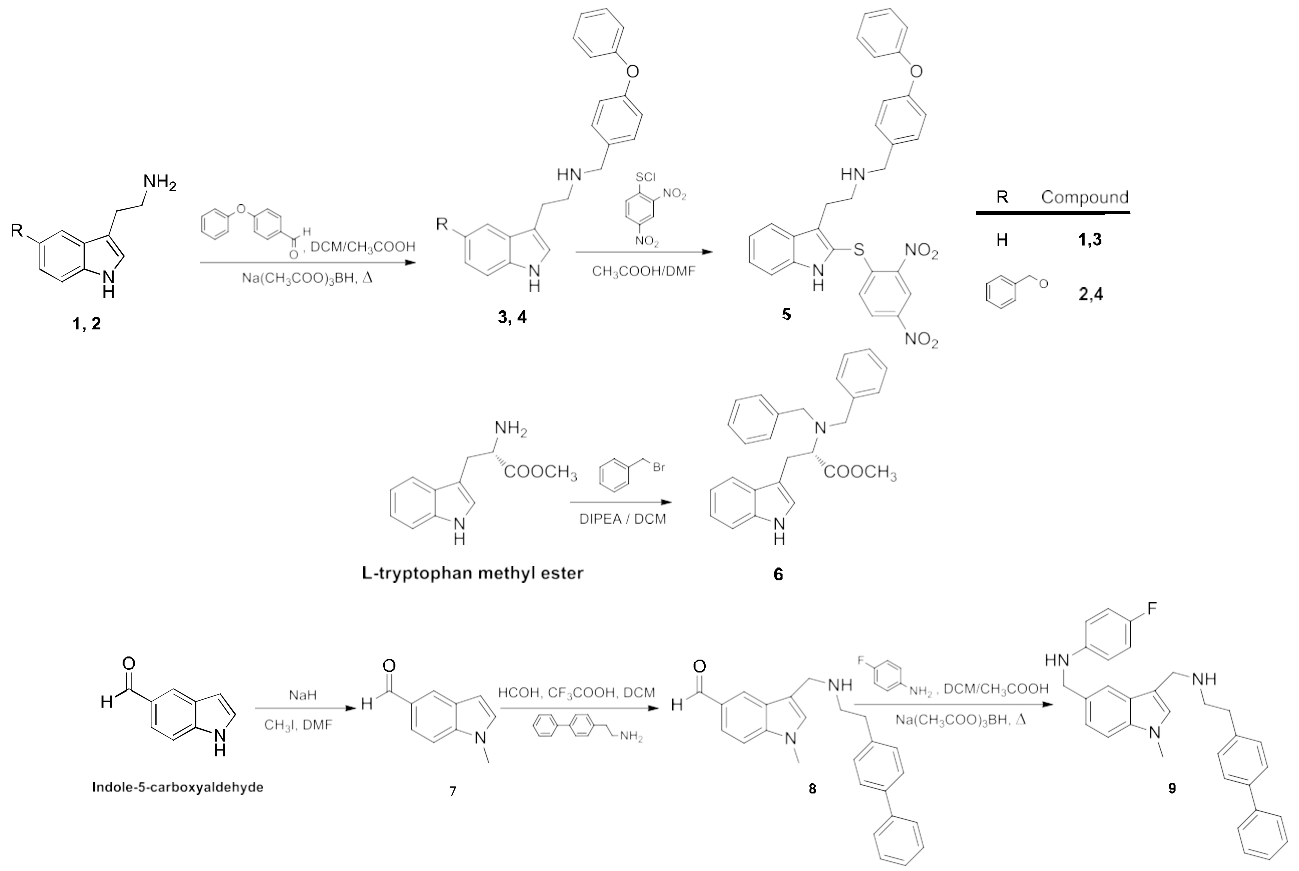

Figure S2: Synthesis of TRPM8 modulators **3**, **4**, **5**, **6** and **9**

Final product **3** was obtained by reductive amination reaction as previously described (reference 30 of the reference list). Compound **4** was synthesized starting from 5-benzyloxytryptamine which was subjected to reductive amination reaction with 4-phenoxybenzaldheyde. Reaction of **3** with 2,4-dinitrosulfenyl chloride in acid media led to the final compound **5.**

For the synthesis of derivative **6**, L-tryptophan methyl ester underwent to a double nucleophilic replacement reaction with benzyl bromide as earlier described (reference 29 of the reference list).

Finally, synthesis of derivative **9** was attained as described earlier (reference 34 of the reference list), starting from indole-5-carboxyaldehyde which was N-methylated by reaction with methyl iodide and sodium hydride. The corresponding N-methyl derivative (**7**) was subjected to a Mannich-

type reaction with formaldehyde and biphenylethyl amine, in acid media, to give intermediate **8**. Reductive amination of 8 using 4-fluoroaniline as reactant, gave **9** as final product.

2-(1H-indol-3-yl)-N-(4-phenoxybenzyl)ethanamine (**3**)

Synthesized starting from tryptamine and 4-phenoxybenzaldehyde in 83% yield as previously described (reference 30 of the reference list). ^{1}H and ^{13}C NMR spectra were in accordance with literature.

2-(5-(benzyloxy)-1H-indol-3-yl)-N-(4-phenoxybenzyl)ethanamine (4)

5-benzyloxytryptamine was reacted with 4-phenoxybenzaldehyde using the same conditions previously described (reference 30 of the reference list). After flash-chromatographic purification compound **4** was isolated in 86% yield. ^{1}H NMR (400 MHz, CD_3OD) δ 3.15 (t, 2H, C*H*$_2$, *J* = 8.0 Hz); 3.31 (t, 2H, C*H*$_2$, *J* = 8.0 Hz); 4.18 (s, 2H, C*H*$_2$); 5.11 (s, 2H, C*H*$_2$); 6.92 (d, 1H, aryl, *J* = 8.0 Hz); 7.03 (t, 3H, aryl, *J* = 8.0 Hz); 7.12-7.20 (m, 3H, aryl); 7.31 (d, 2H, aryl, *J* = 8.0 Hz); 7.33 (s, 1H, aryl); 7.35 (t, 2H, aryl, *J* = 8.0 Hz); 7.38-7.49 (m, 7H, aryl); ^{13}C NMR (100 MHz, CD_3OD) δ 22.0; 48.2; 50.3; 70.8; 101.6; 111.9; 112.3; 118.3; 119.1; 123.6; 123.7; 123.8; 127.0; 127.3; 127.4; 128.0; 129.7; 131.3; 131.4; 132.4; 137.9; 152.9. ESI-MS m/z calcd for $C_{30}H_{28}N_2O_2$, 448.56; found 448.60.

2-(2-((2,4-dinitrophenyl)thio)-1H-indol-3-yl)-N-(4-phenoxybenzyl)ethanamine (5)

146 mg of compound **3** (0.41 mmol) were dissolved in 10 mL of CH_3COOH/DMF (8:2 v:v) and maintained under magnetic stirring. To this solution 192 mg (0.82 mmol) of 2,4-dinitrophenylsulphenyl chloride were added. The mixture was allowed to react at room temperature under nitrogen stream for 2 hours. Then, a saturated solution of $NaHCO_3$ was added and the pH adjusted to 8. The resulting aqueous phase was extracted two times with dichloromethane. Organic phases were collected, dried over Na_2SO_4, filtered and evaporated in vacuo. Crude product was purified by flash chromatography giving **5** as a yellowish solid in 78% yield.

^{1}H NMR (400 MHz, $CDCl_3$) δ 2.89 (t, 2H, C*H*$_2$, *J* = 8.0 Hz); 3.03 (t, 2H, C*H*$_2$, *J* = 8.0 Hz); 3.71 (s, 2H, C*H*$_2$); 4.62 (s, 1H, NH); 6.76 (t, 3H, aryl, *J* = 8.0 Hz); 6.87 (d, 2H, aryl, *J* = 8.0 Hz); 7.01 (t, 1H, aryl, *J* = 8.0 Hz); 7.08-7.18 (m, 3H, aryl); 7.23 (t, 3H, aryl, *J* = 8.0 Hz); 7.29 (d, 1H, aryl, *J* = 8.0 Hz); 7.59 (d, 1H, aryl, *J* = 8.0 Hz); 7.91 (d, 1H, aryl, *J* = 8.0 Hz); 8.69 (s, 1H, NH); 8.93 (s, 1H, aryl); ^{13}C NMR (100 MHz, $CDCl_3$) δ 24.5; 48.3; 52.0; 111.7; 118.4; 118.8; 118.9; 119.9; 120.7; 121.4; 123.4; 123.7; 125.0; 127.2; 127.5; 128.8; 129.7; 129.9; 132.1; 137.9; 143.9; 144.8; 146.5; 156.7; 157.0; 176.4. ESI-MS m/z calcd for $C_{29}H_{24}N_4O_5S$, 540.15; found 540.18.

(S)-methyl 2-(dibenzylamino)-3-(1H-indol-3-yl)propanoate (6)

Compound **6** was obtained by nucleophilic substitution of L-tryptophan methyl ester using an excess of (bromomethyl)benzene in the same conditions described previously (reference 29 of the reference list). ^{1}H and ^{13}C NMR spectra were in accordance with literature.

1-methyl-1H-indole-5-carbaldehyde (7)

Intermediate **7** was synthesized in 89% of yield starting from indole-5-carboxyaldehyde and methyl iodide, using NaH as base as previously described (reference 34 of the reference list). ^{1}H and ^{13}C NMR spectra were in accordance with literature.

3-(((2-([1,1'-biphenyl]-4-yl)ethyl)amino)methyl)-1-methyl-1H-indole-5-carbaldehyde (8)

Synthesized from **7** by reaction with formaldehyde and 4-biphenylethyl amine using trifluoroacetic acid as catalyst as described before (reference 34 of the reference list). Compound **8** was isolated in 70% yield after flash chromatography. ^{1}H and ^{13}C NMR spectra were in accordance with literature.

N-((3-(((2-([1,1'-biphenyl]-4-yl)ethyl)amino)methyl)-1-methyl-1H-indol-5-yl)methyl)-4-fluoroaniline (9)

Compound **9** was synthesized by reaction of **8** was with 4-fluoroaniline under the conditions described elsewhere (reference 34 of the reference list). The crude product was purified by column chromatography using mixtures of DCM/MeOH as eluent leading to the corresponding 3,5-bisaminomethyl indole derivative **4** in 63% of yield.

^{1}H and ^{13}C NMR spectra were in accordance with literature.

Table S1. In vitro pharmacological characterization for synthesized compounds

Comp.	Structure	% Efficacy	Potency (nM)
**3**	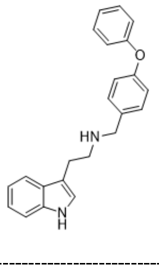	100.0 ±8.4 [a]	EC_{50}=12.30±4.21
**4**	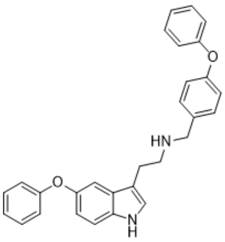	69.0±7.2 [b]	IC_{50}=204±15
**5**	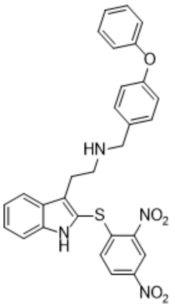	59.5±6.7 [b]	IC_{50}=340±26
**6**	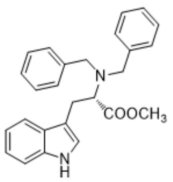	100.0±2.4 [c]	IC_{50}=0.2±0.2
**9**	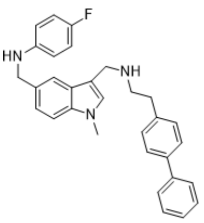	59.4±9.7 [b]	IC_{50}=5000±285

[a] maximal currents at 5 μM compared to L-menthol 100 μM [b] Inhibition 300 μM menthol-evoked currents at 300 nM compounds [c] Inhibition 100 μM menthol-evoked currents at 5 μM compounds

Figure S3

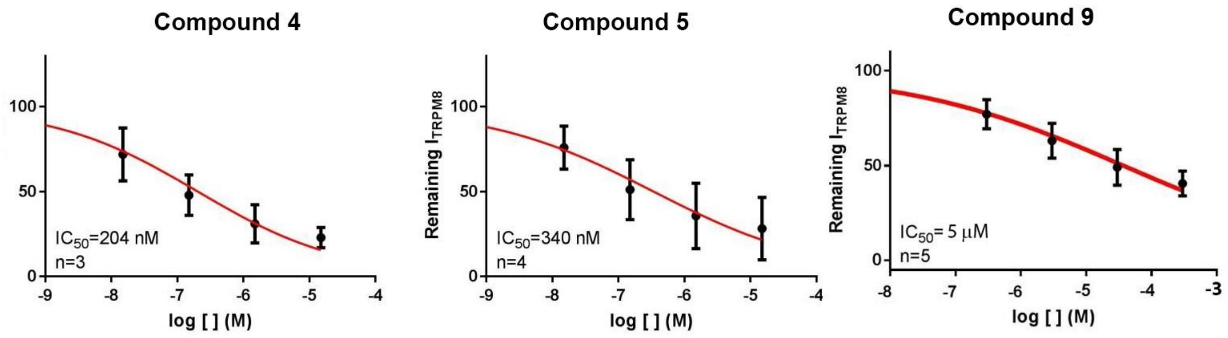

Figure S3: Concentration–response curves for menthol evoked TRPM8 current inhibition by compounds **4**, **5** and **9**.

Figure S4

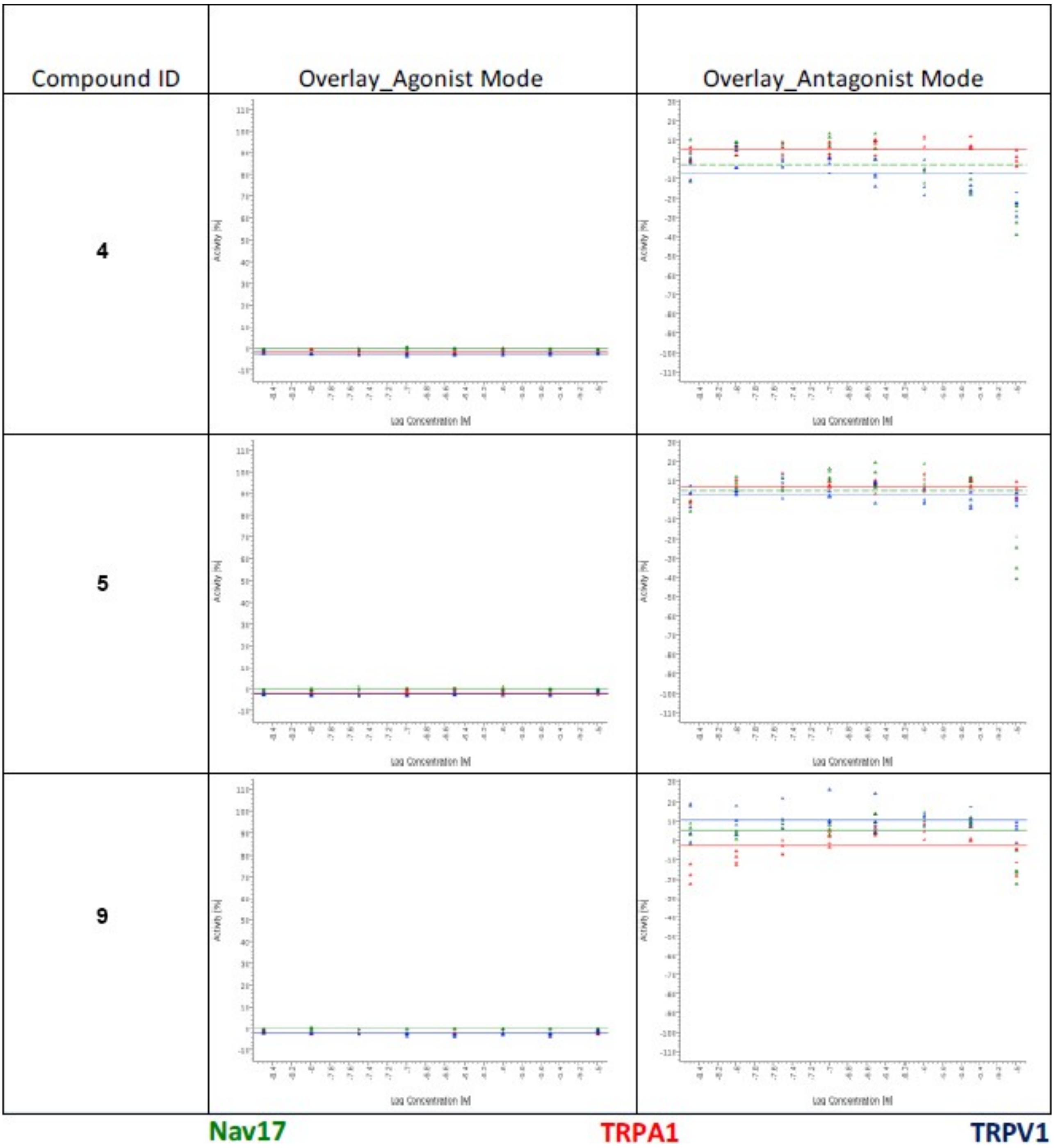

Figure S4: Effects of compounds **4**, **5** and **9** over $NaV_{1.7}$, TRPA1 and TRPV1 channels.

Figure S5

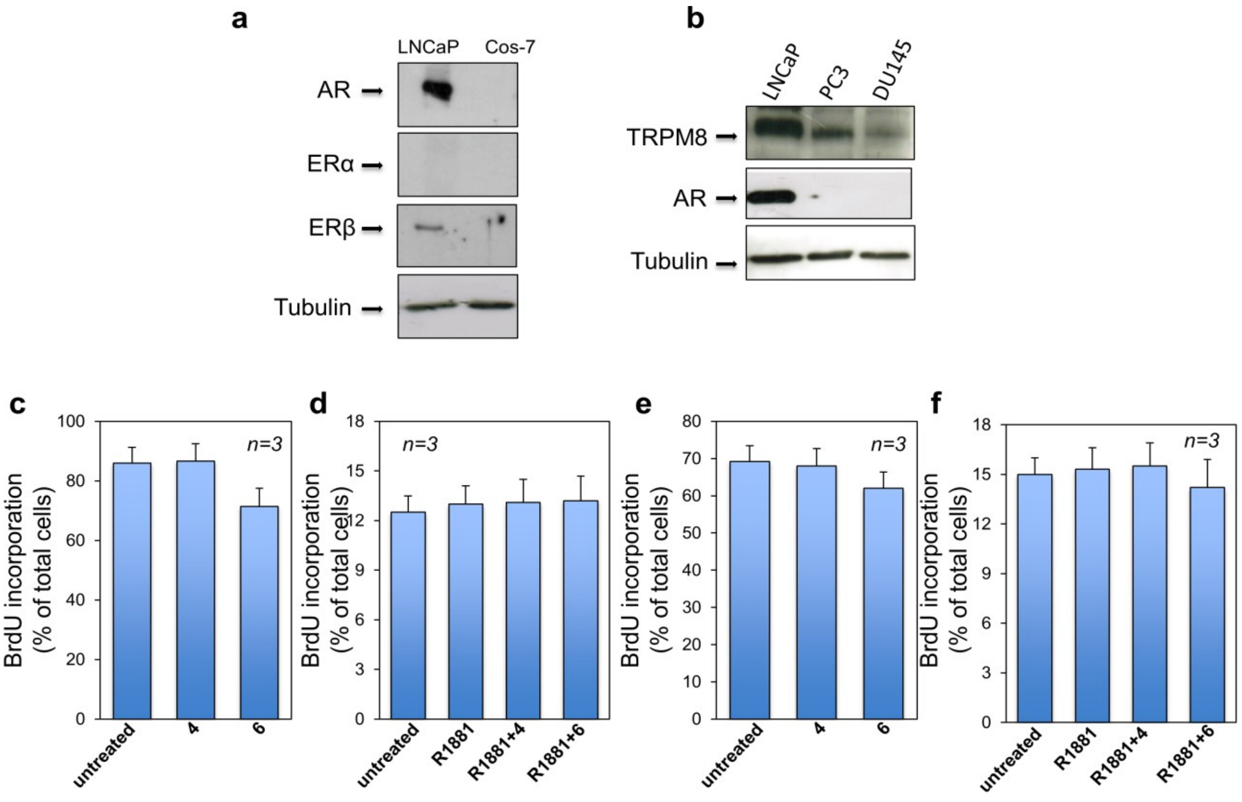

Figure S5. Expression of steroid receptors (a) and TRPM8 (b) in PC-derived cell lines. Effect of TRPM8 antagonists on serum- or androgen-stimulated DNA synthesis in AR-negative PC cells (C-F).

In **a** and **b**, lysate proteins prepared from the indicated cell lines were analyzed by WB technique, using the antibodies against the indicated proteins. In **a**, Cos-7 cells were used as negative control, as they do not express sex steroid receptors (see Ref. 33 in the Reference's section). In **b**, LNCaP cells were used as positive control for AR expression, while PC3 and DU145 cells were employed as negative control, since they do not express AR (see Ref. 26 in the Reference's list). In **a** and **b**, WB are representative of three different experiments. Densitometric analysis from these experiments was also calculated and data are presented below in this file (Supplementary Information file). Cycling PC3 (**c**) and DU145 (**e**) cells, or quiescent PC3 (**d**) and DU145 (**f**) cells were plated on coverslips and then left untreated or treated for 18h with the indicated compounds. The androgen, R1881 was added at 10 nM. Compounds **4** and **6** were used at 1 μM. After *in vivo* pulse with 100 μM BrdU, its incorporation into newly synthesized DNA was analyzed by IF and expressed as % of total cells. Means and standard errors (SEMs) are shown. *n,* represents the number of experiments.

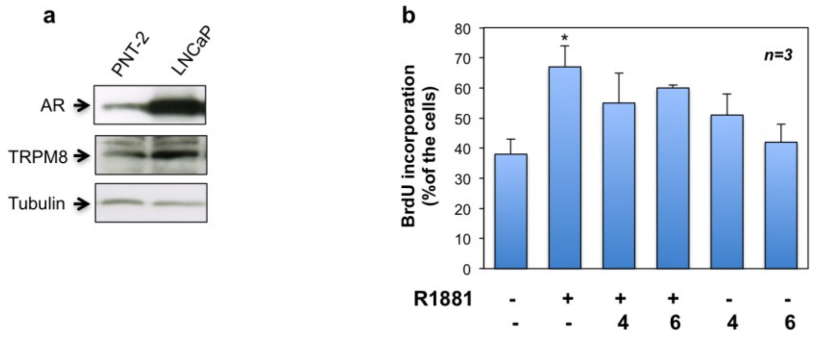

Figure S6. Expression of AR and TRPM8 in normal epithelial prostate PNT-2 cells (a) and effect of TRPM8 antagonists on the androgen-stimulated DNA synthesis in PNT-2 cells (b).

In **a**, lysate proteins prepared from the indicated cell lines were analyzed by WB technique, using the antibodies against the indicated proteins. In **a**, WB are representative of three different experiments. Densitometric analysis from these experiments was also calculated and data are presented below in this file (Supplementary Information file). Quiescent PNT-2 cells (**b**) were plated on coverslips and then left untreated or treated for 18h with the indicated compounds. R1881 was added at 10 nM, while the compounds 4 and 6 were used at 1 µM. After in vivo pulse with 100 µM BrdU, its incorporation into newly synthesized DNA was analyzed by IF and expressed as % of total cells. Means and standard errors (SEMs) are shown. *n,* represents the number of experiments.

Figure S7

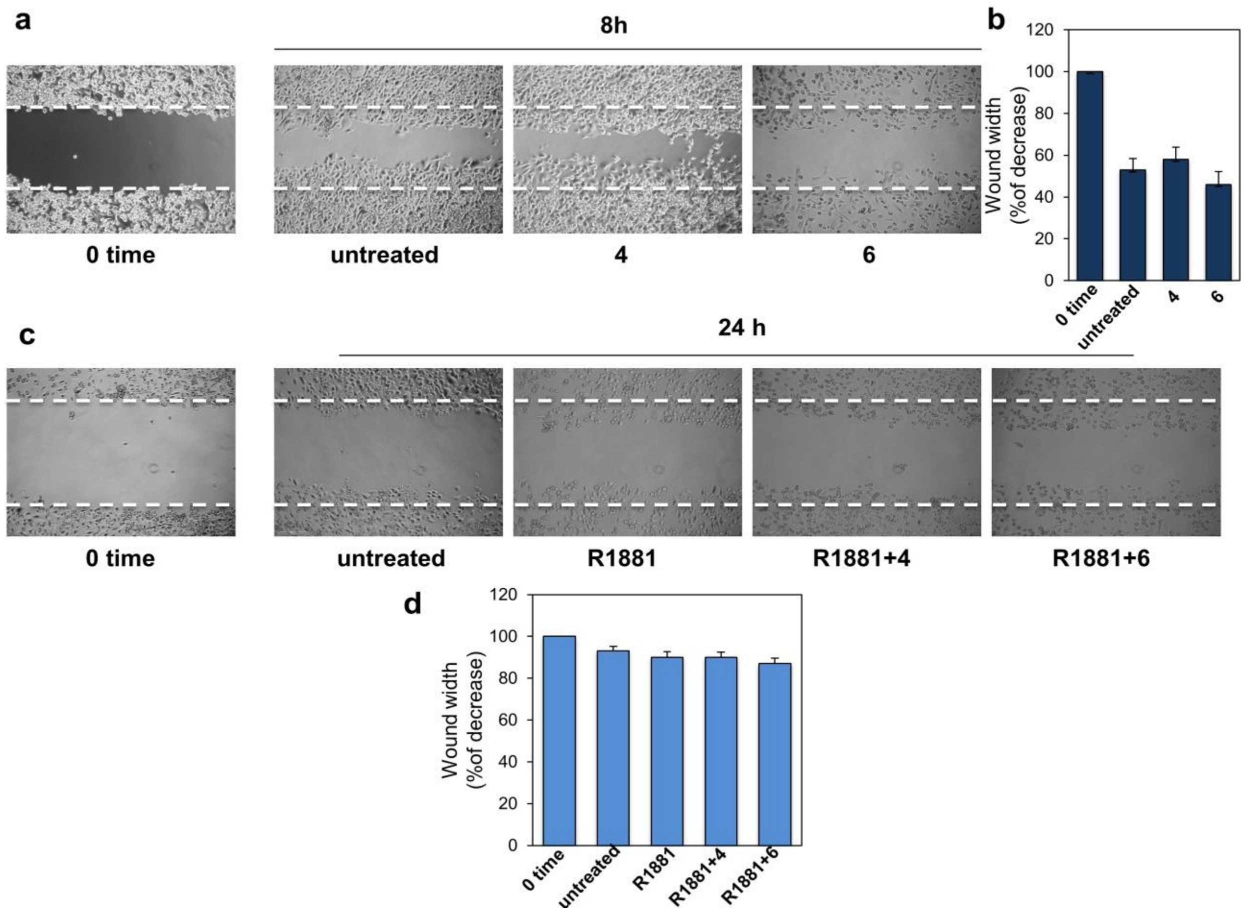

Figure S7. TRPM8 antagonists do not affect the motility of AR-negative PC3 cells. Cycling (**a**) or quiescent (**c**) PC3 cells were scratch-wounded and then left untreated or treated with the indicated compounds. R1881 was added at 10 nM. The compounds **4** or **6** were added at 1 μM. Cells were allowed to migrate for the indicated times. Phase-contrast images in **a** and **c** are representative of three different experiments, each in duplicate. In **b** and **d**, the wound area was measured using the Leica Suite Software. Data are presented as % in wound-width decrease over the control cells, analyzed at 0 time. Means and standard errors (SEMs) are shown.

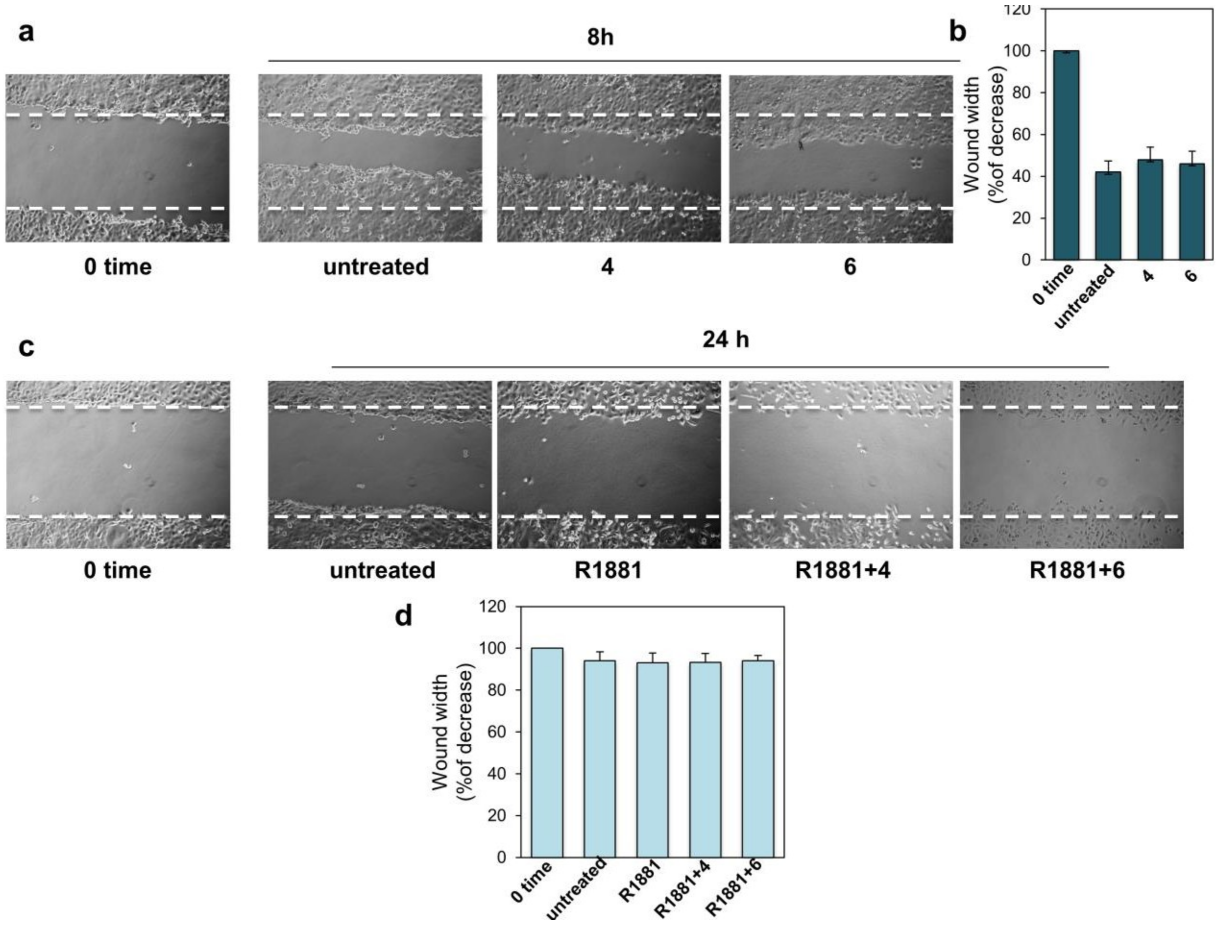

Figure S8. TRPM8 antagonists do not affect the motility of AR-negative DU145 cells. Cycling (**a**) or quiescent (**c**) DU145 cells were wounded and left untreated or treated with the indicated compounds. R1881 was used at 10 nM and the compounds **4** or **6** at 1 μM. Cells were allowed to migrate for the indicated times. Phase-contrast images in **a** and **c** are representative of three different experiments, each in duplicate. In **b** and **d**, the wound area was measured using the Leica Suite Software. Data are presented as % in wound-width decrease over the control cells, analyzed at 0 time. Means and standard errors (SEMs) are shown.

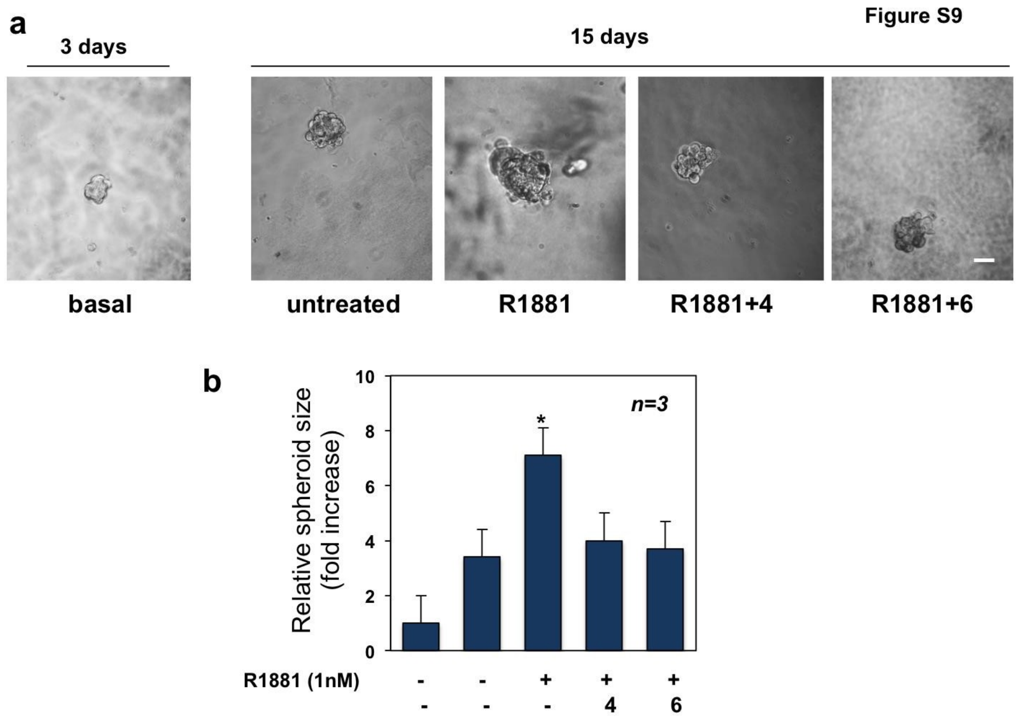

Figure S9. TRPM8 antagonists inhibit the increase in LNCaP cell spheroid/organoid size induced by a low ligand concentration (a and b). In **a**, a 3D structure was observed in LNCaP cells after 3 days of culture. At that time, the organoids were left untreated or treated with 1 nM R1881 in absence or presence of TRPM8 antagonists 4 and 6 (both used at 1µM). Changes in dimension and structure of organoids were monitored for additional 15 days. Phase-contrast microscopy is shown in (**a**). Scale bar, 100 µm. In **b**, the spheroid size was calculated and quantified as described in Methods. It was expressed as fold increase in the relative spheroid size. Means and SEMs from three different experiments are shown. * $p < 0.05$ for the indicated experimental points versus the corresponding untreated control. *n,* represents the number of experiments.

Western Blots for Review - images of original western blots

Figure 1

Figure 1a

TRPM8

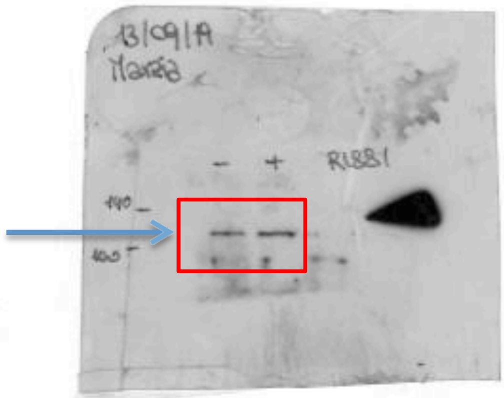

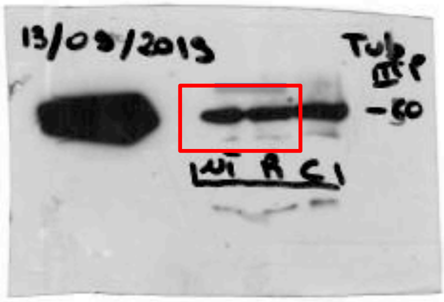

Figure 1a Tubulin

Quantification of Western blots presented in Figure 1a

	TRPM8/Tubulin (fold change) n=3	SD
Unstimulated	1	
R1881	1,4	0,098

Figure 2

Figure 2c cyclin D1 compound 4

Cyclin D1

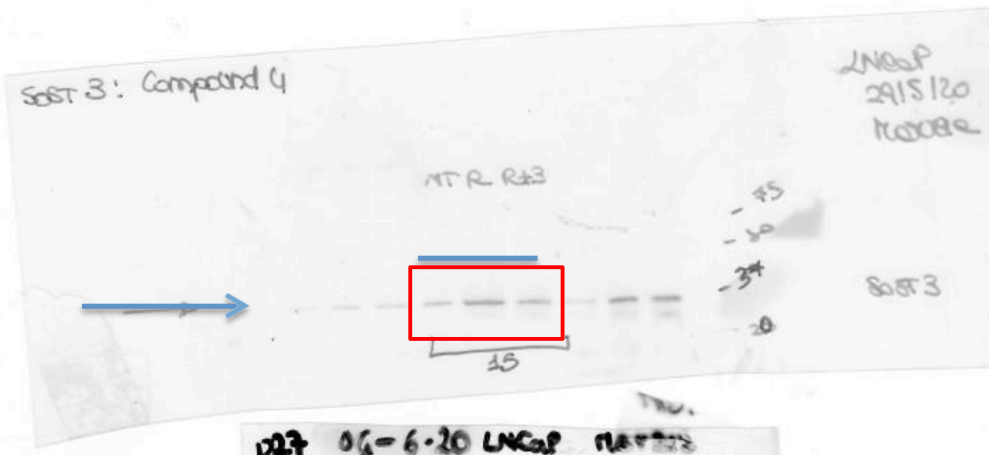

Figure 2c p27 compound 4

p27

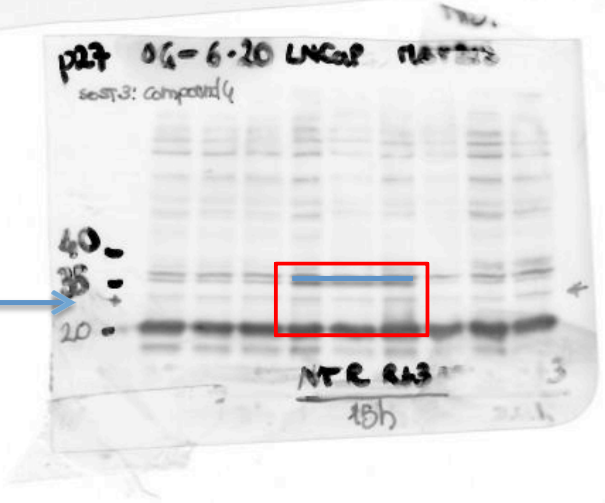

Figure 2c Cdk4 compound 4

Cdk4

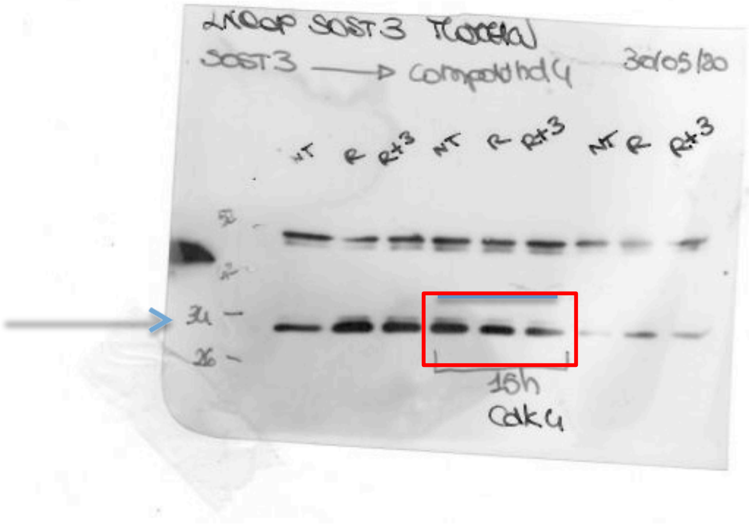

Figure 2d cyclin D1
Compound 6

Cyclin D1

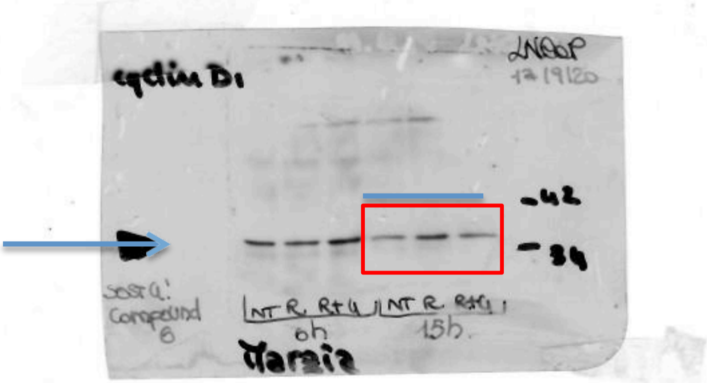

p27

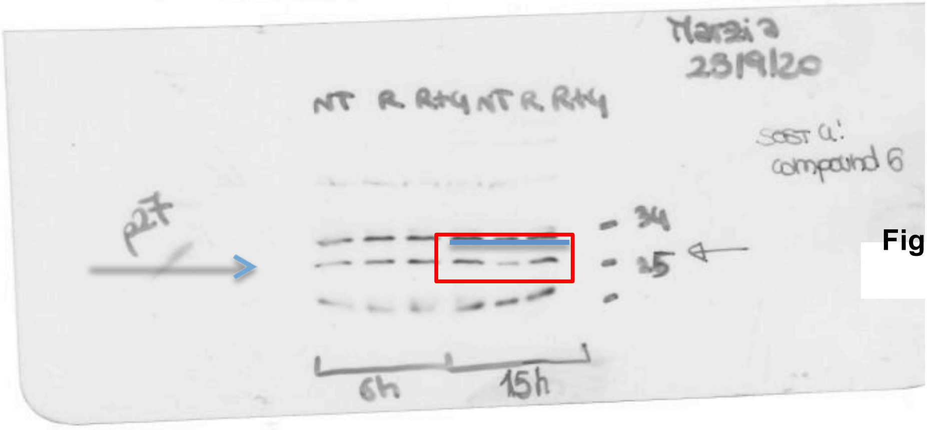

Figure 2d p27 compound 6

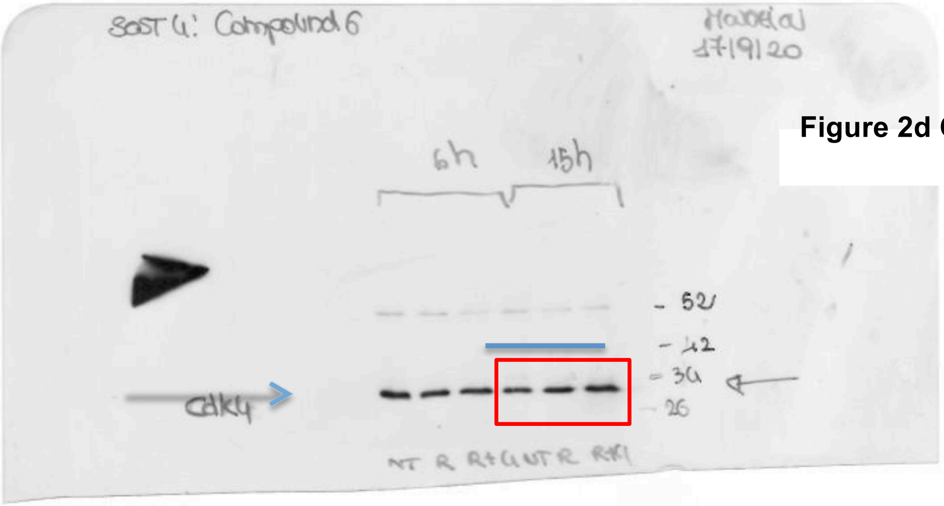

Figure 2d Cdk4 compound 6

Cdk4

Cyclin D1

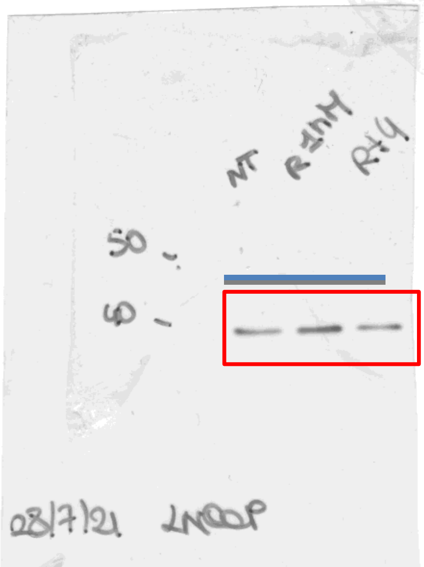

Figure 2e cyclin D1 compound 4

p27

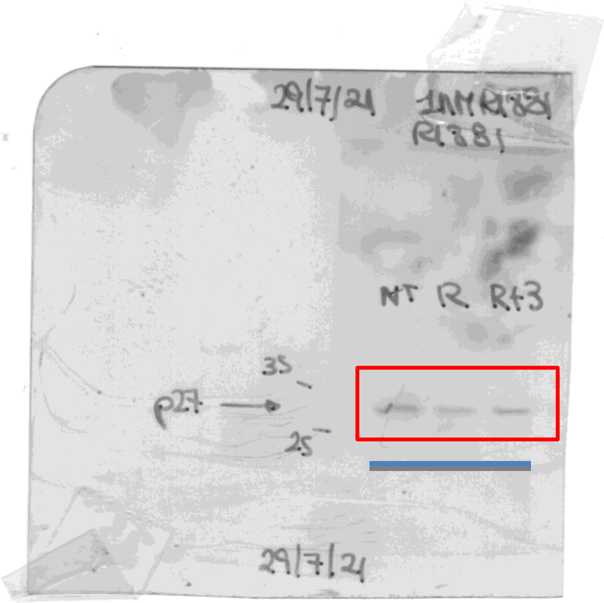

Figure 2e p27 compound 4

Cdk4

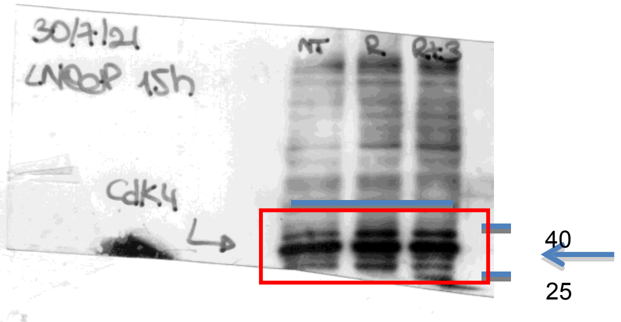

Figure 2e Cdk4 compound 4

Cyclin D1

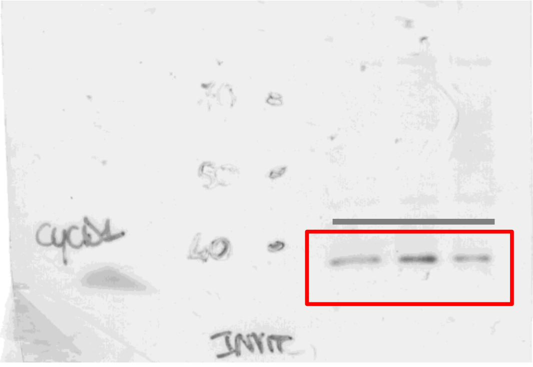

Figure 2f cyclin D1 compound 6

p27

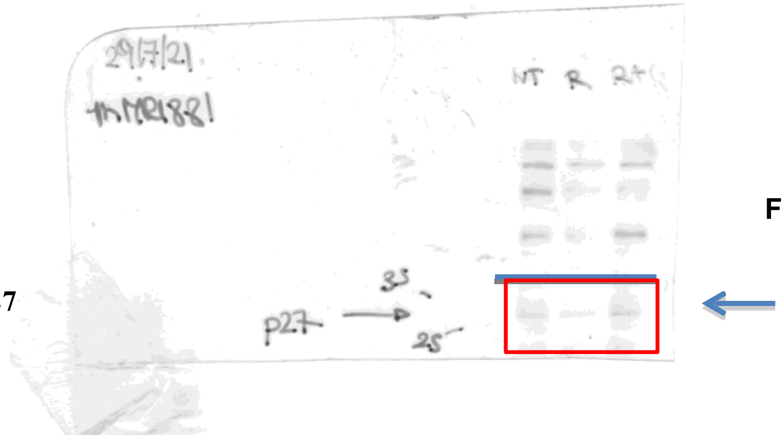

Figure 2f p27 compound 6

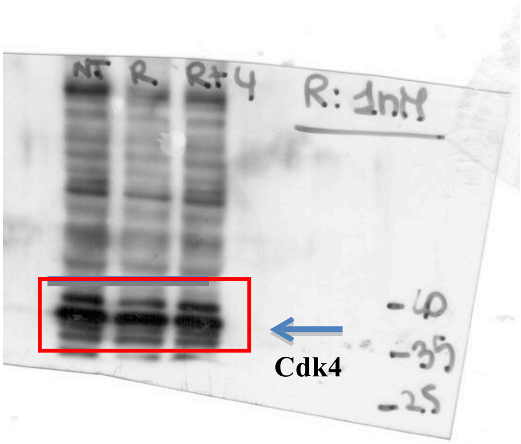

Cdk4

Figure 2f cdk4 compound 6

Quantification of Western blots presented in Figure 2c

		fold increase over the basal level n=3	SD
Cyclin D1	Unstimulated	1	
	R1881	2,5	0,06
	R1881 + 4	1,3	0,04
P27	Unstimulated	1	
	R1881	0,4	0,01
	R1881 + 4	1,2	0,05
Cdk4	Unstimulated	1	
	R1881	0,9	0,04
	R1881 + 4	0,87	0,05

Quantification of Western blots presented in Figure 2d

		fold increase over the basal level n=3	SD
Cyclin D1	Unstimulated	1	
	R1881	2,7	0,05
	R1881 + 6	1,2	0,05
P27	Unstimulated	1	
	R1881	0,4	0,02
	R1881 + 6	1,1	0,04
Cdk4	Unstimulated	1	
	R1881	1	0,05
	R1881 + 6	1,1	0,04

Quantification of Western blots presented in Figure 2e

		fold increase over the basal level n=3	SD
Cyclin D1	Unstimulated	1	
	R1881	1,5	0,05
	R1881 + 4	0,9	0,05
P27	Unstimulated	1	
	R1881	0,6	0,02
	R1881 + 4	1,1	0,04
Cdk4	Unstimulated	1	
	R1881	1	0,02
	R1881 + 4	1	0,02

Quantification of Western blots presented in Figure 2f

		fold increase over the basal level n=3	SD
Cyclin D1	Unstimulated	1	
	R1881	1,7	0,08
	R1881 + 6	0,9	0,05
P27	Unstimulated	1	
	R1881	0,6	0,03
	R1881 + 6	1	0,05
Cdk4	Unstimulated	1	
	R1881	1,1	0,03
	R1881 + 6	1,1	0,07

Figure 3 a

TRPM8

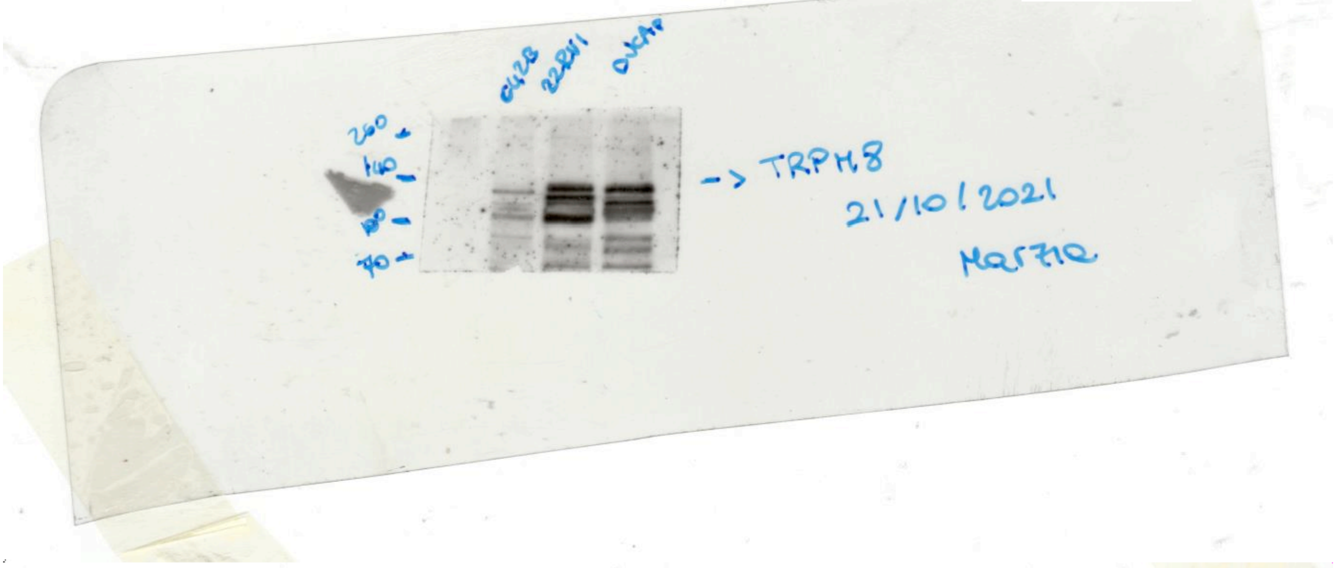

AR

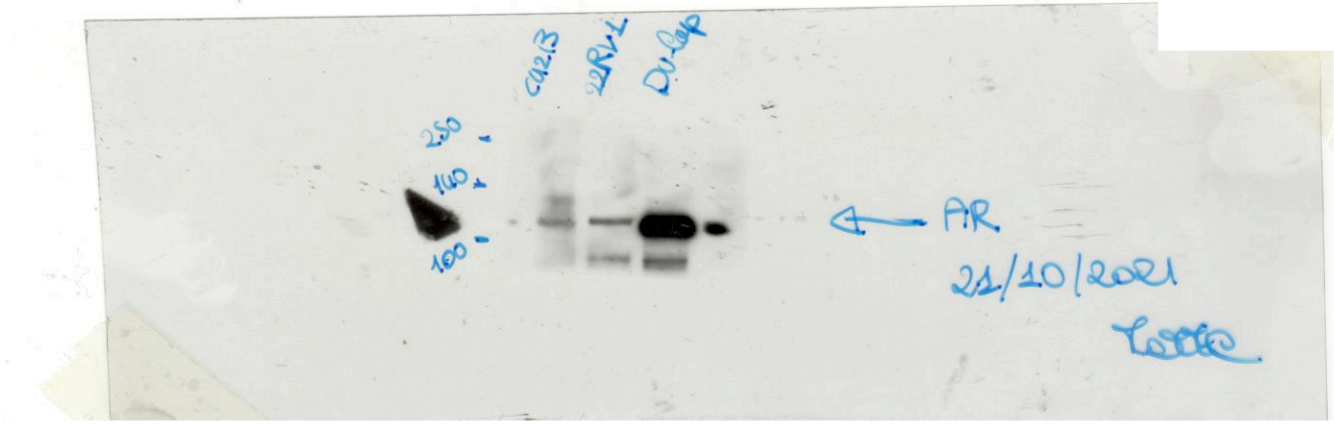

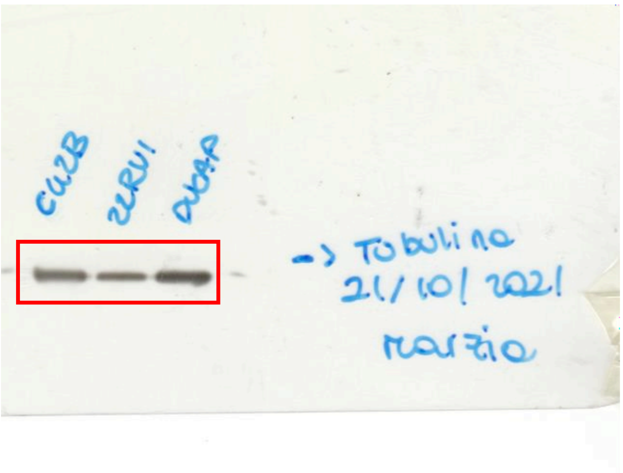

Quantification of Western blots presented in Figure 3 a

		fold increase over the basal level n=3	SD
AR/tubulin ratio	C4-2B	1	
	22Rv1	2,1	0,06
	DU-CaP	12,7	0,03
TRPM8/Tubulin ratio	C4-2B	1	
	22Rv1	6,2	0,02
	DU-CaP	5,6	0,04

Figure 6

Figure 6b, right panel

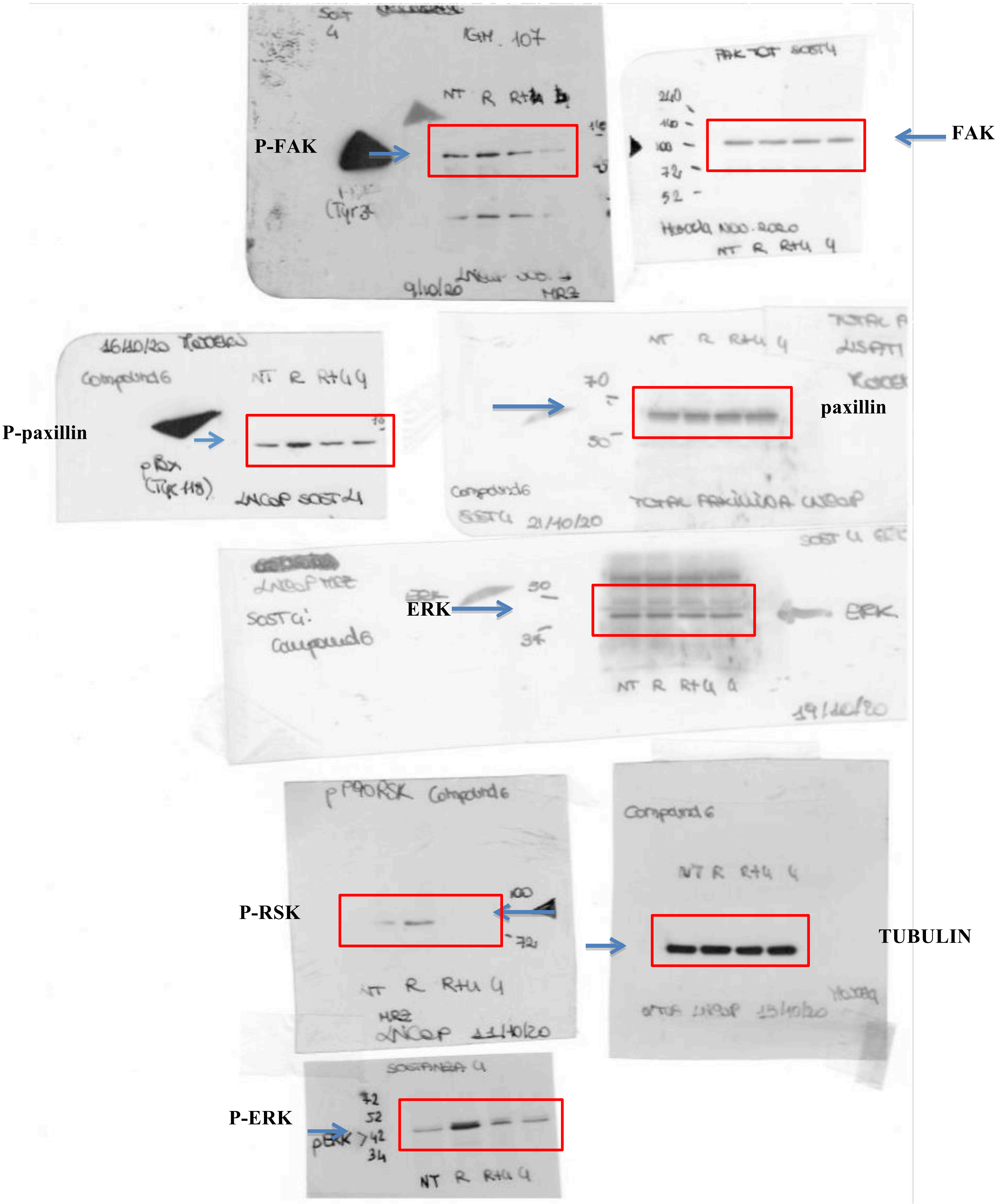

Figure 6b, Left panel

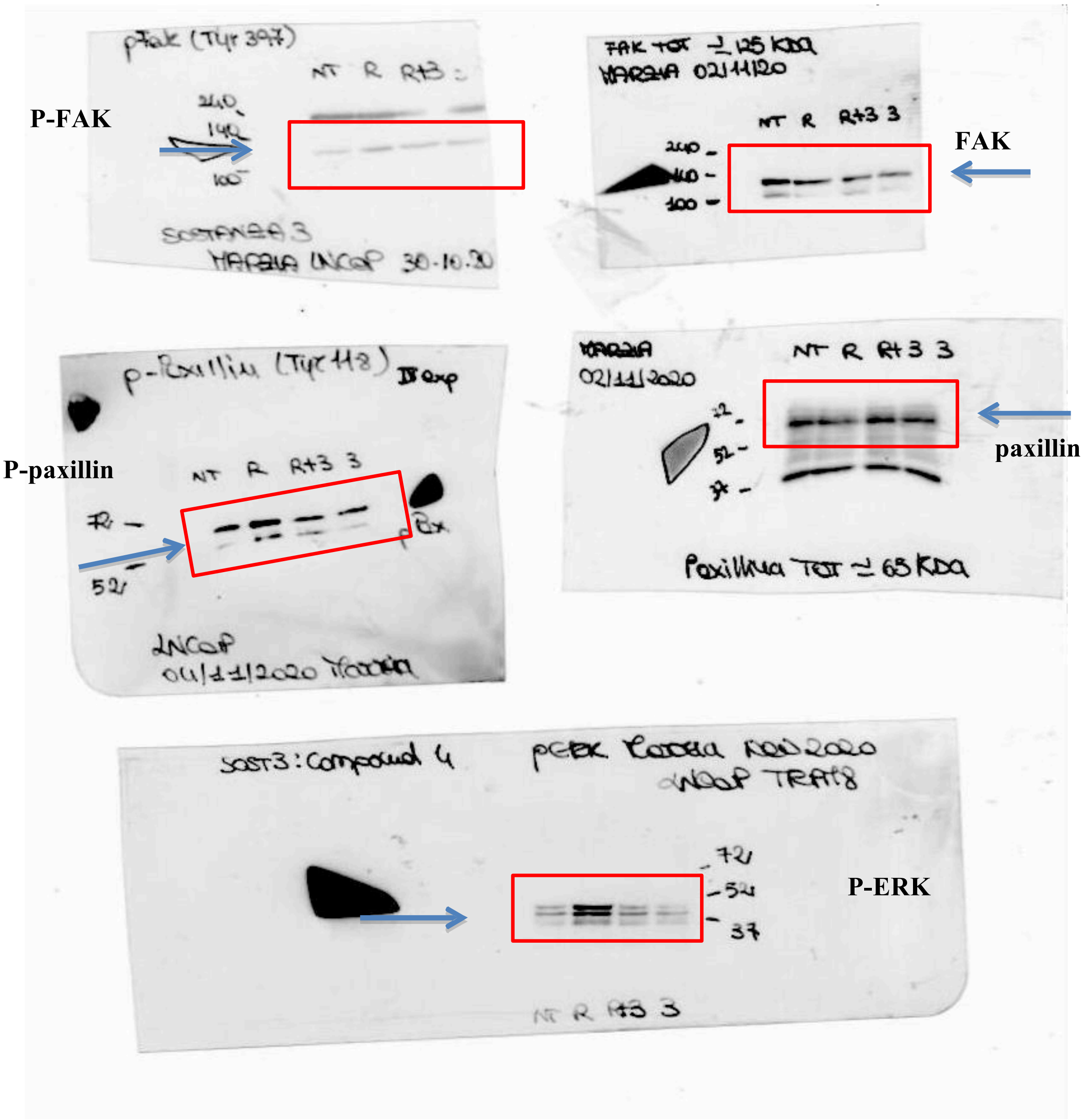

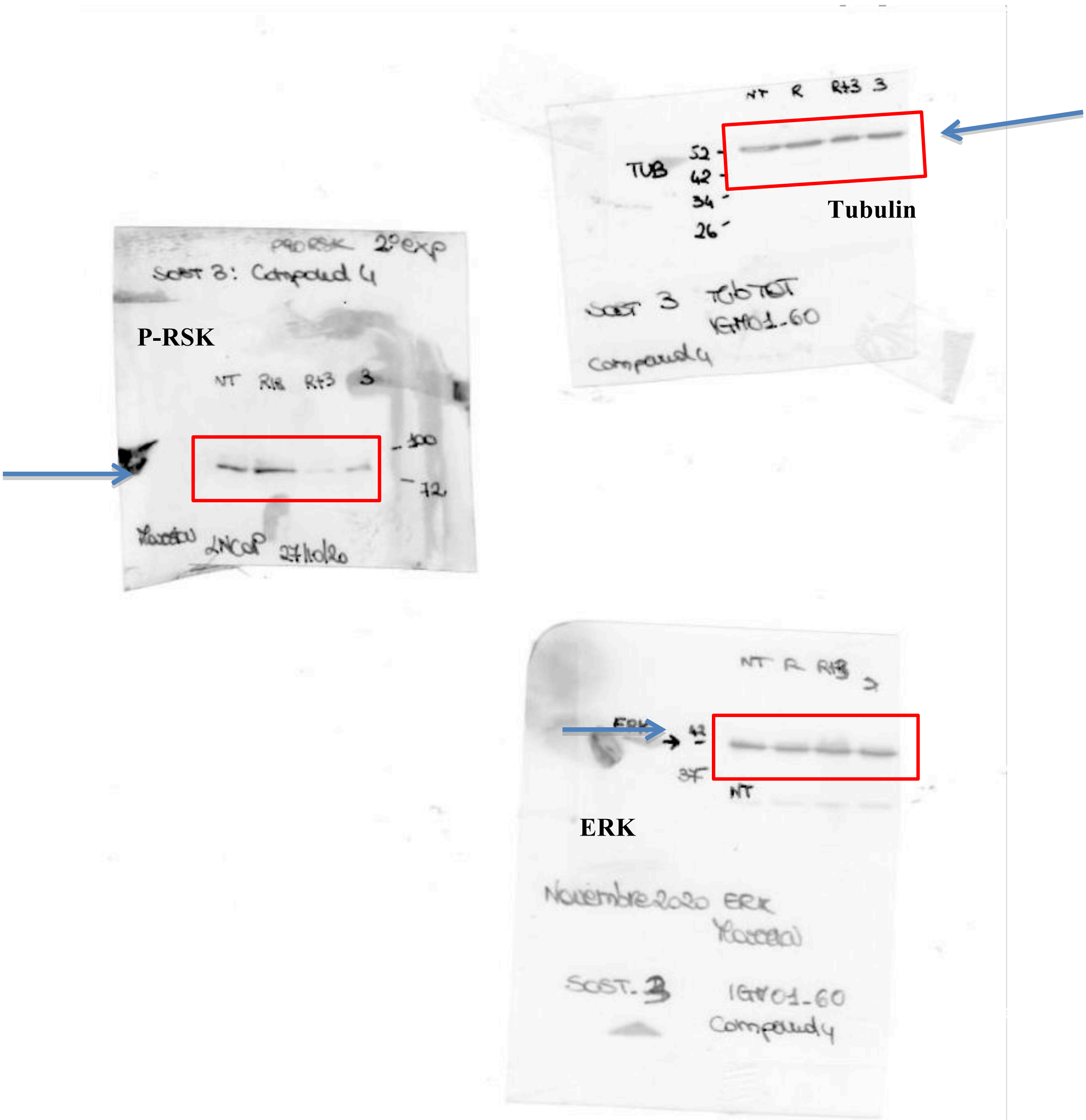
Tubulin
P-RSK
ERK
NT R R+3 3
TUB
52
42
34
26
SOST 3
Compound 4
SOST 3: Compound 4
NT
R+3
3
100
72
LNCaP
27/10/20
ERK
42
37
Novembre 2020 ERK
SOST. 3
IGM01-60
Compound 4

Figure 6 c

FAK

P-FAK

paxillin

P-paxillin

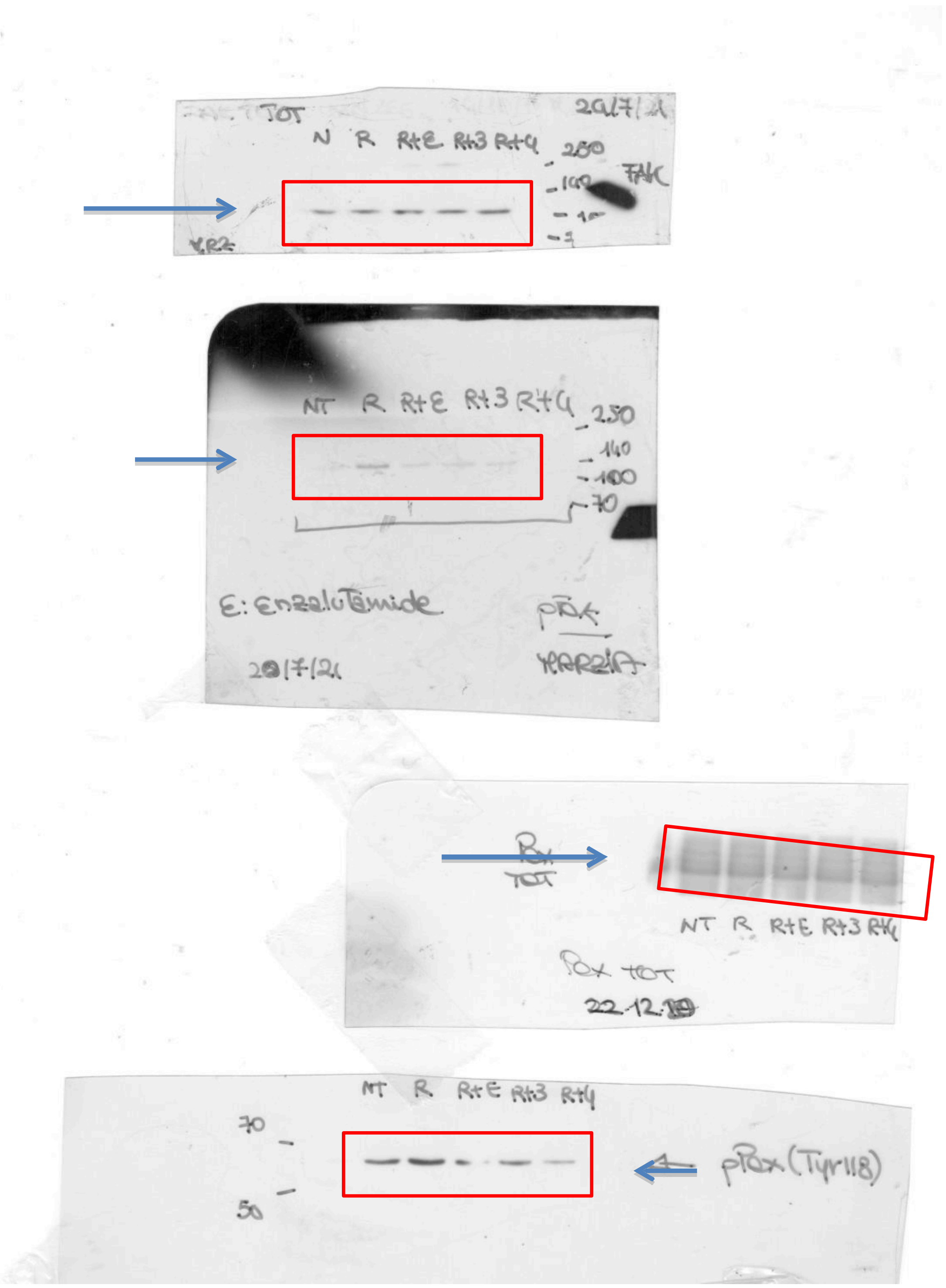

P-ERK

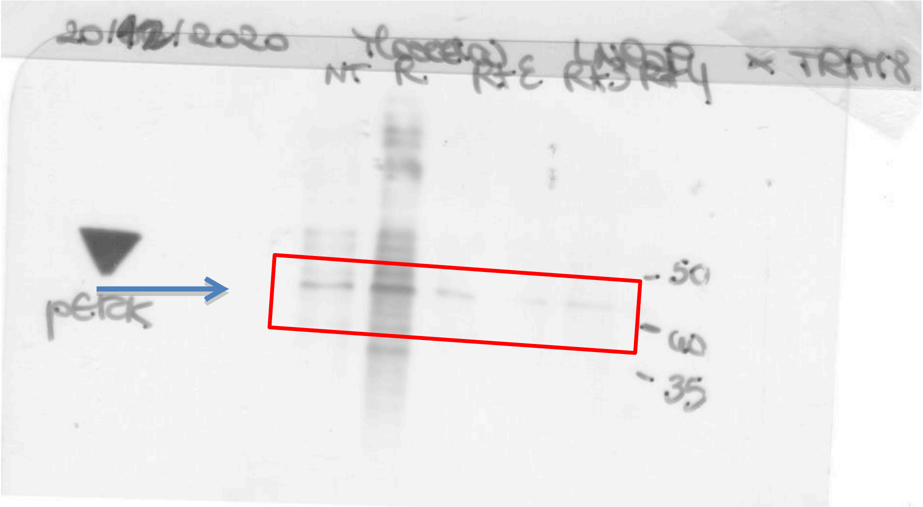

ERK

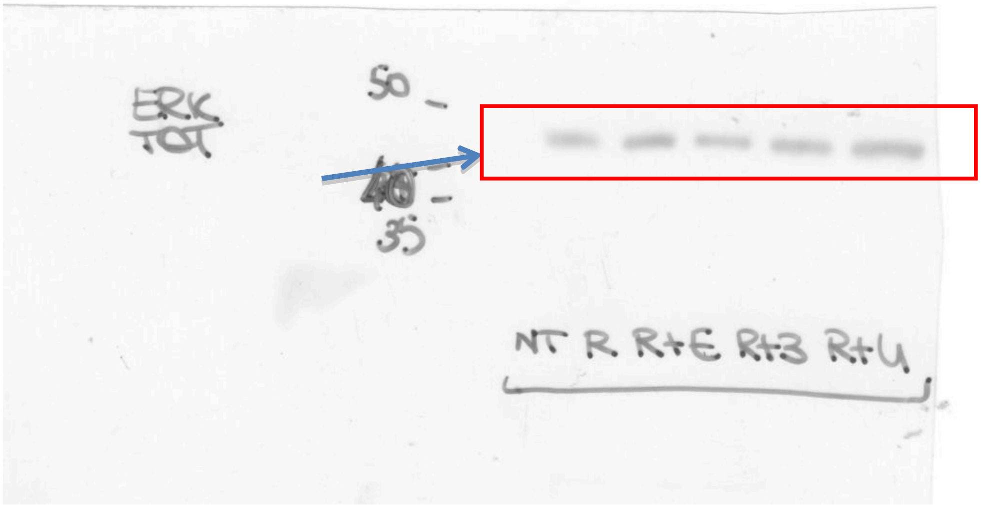

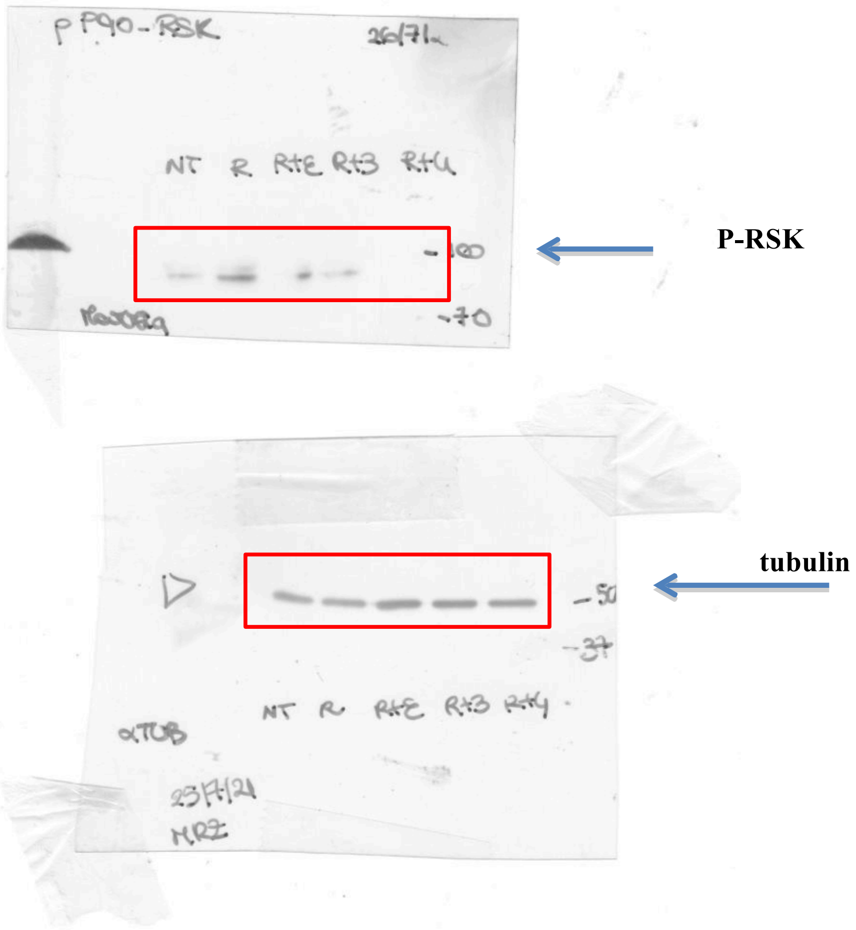
P-RSK
tubulin

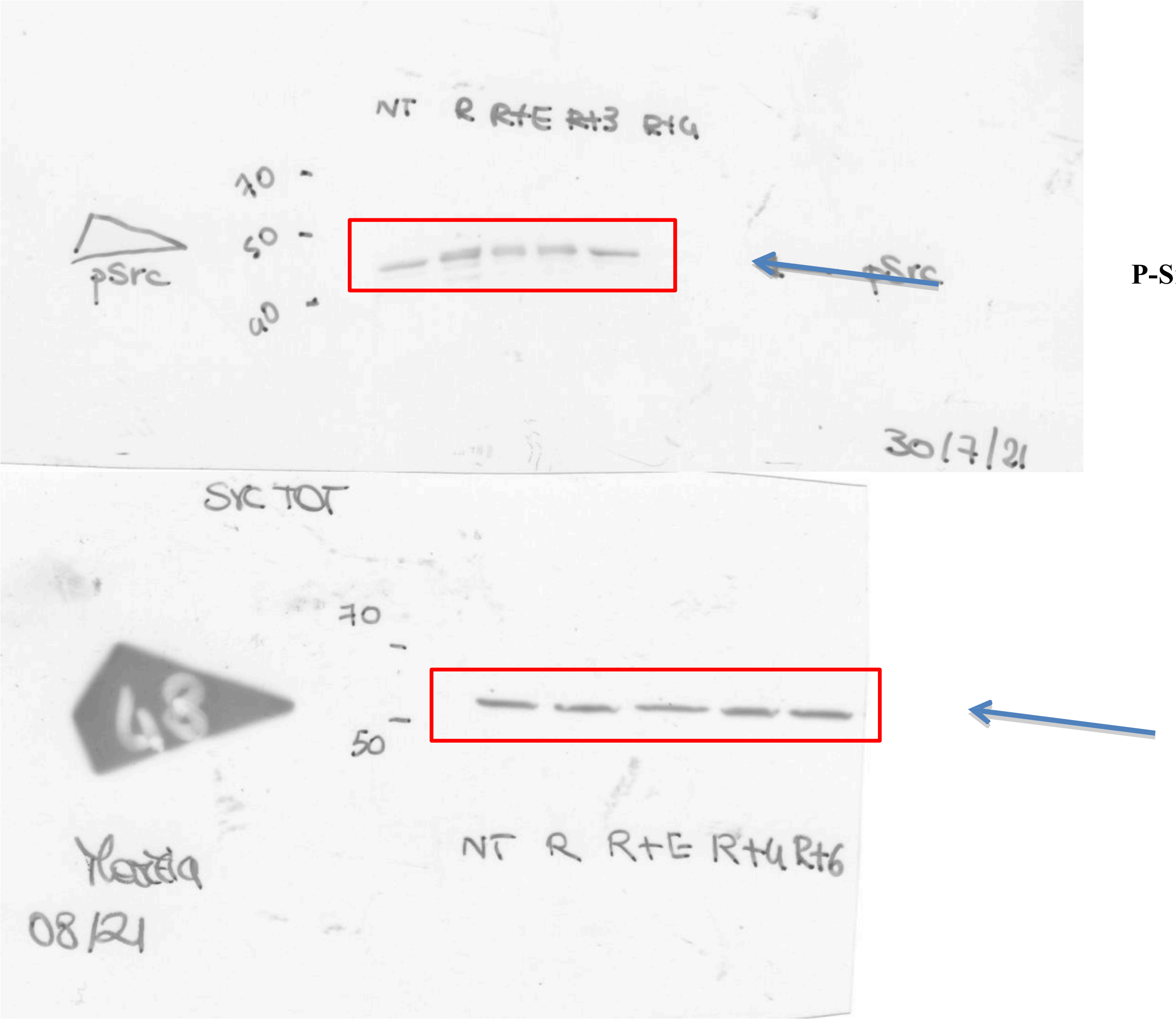

P-Src

Src

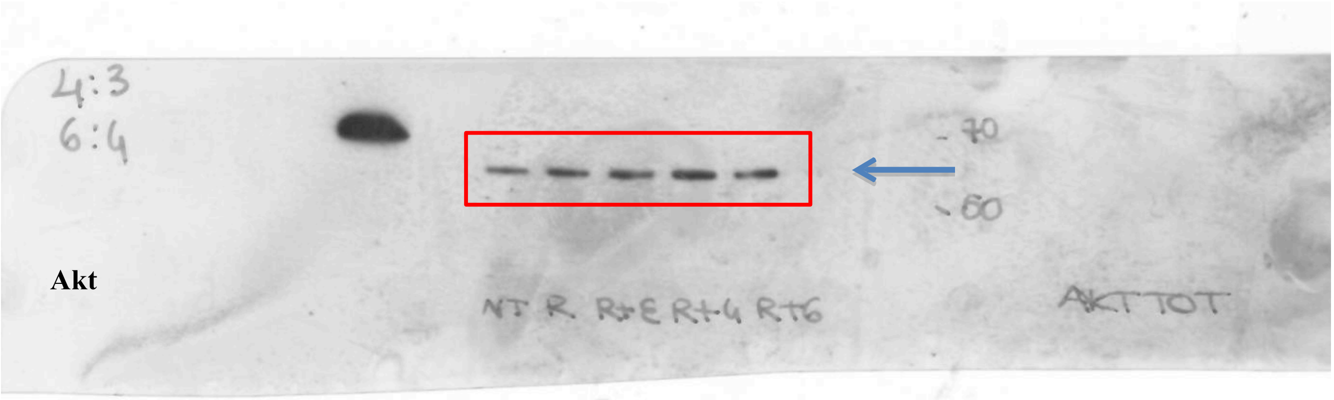

Akt

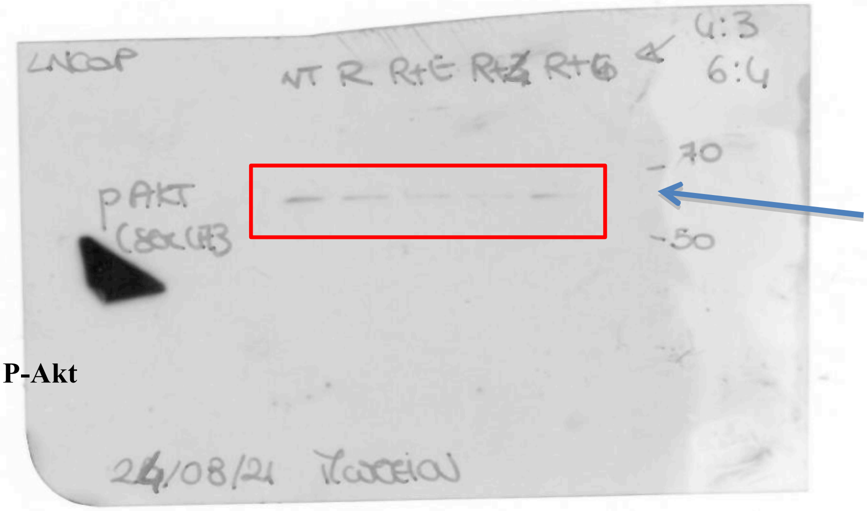

P-Akt

Quantification of Western blots presented in Figure 6b left panel

		fold increase over the basal level n=3	SD
FAK	Unstimulated	1	
	R1881	0,8	0,08
	R1881 + 4	0,8	0,05
	4	0,9	0,04
P-FAK	Unstimulated	1	
	R1881	2	0,02
	R1881 + 4	0,8	0,02
	4	1	0,03
paxillin	Unstimulated	1	
	R1881	0,9	0,08
	R1881 + 4	1	0,05
	4	0,9	0,04
P-paxillin	Unstimulated	1	
	R1881	3	0,09
	R1881 + 4	1	0,07
	4	1	0,07
ERK	Unstimulated	1	
	R1881	0,9	0,02
	R1881 + 4	1	0,02
	4	1	0,03
P-ERK	Unstimulated	1	
	R1881	3,9	0,2
	R1881 + 4	1,3	0,09
	4	0,6	0,03
P-RSK	Unstimulated	1	
	R1881	2,3	0,3
	R1881 + 4	0,5	0,07
	4	0,7	0,07
tubulin	Unstimulated	1	
	R1881	1	0,02
	R1881 + 4	1	0,04
	4	1	0,03

Quantification of Western blots presented in Figure 6b right panel

		fold increase over the basal level n=3	SD
FAK	Unstimulated	1	
	R1881	1	0,03
	R1881 + 6	1	0,02
	6	1	0,03
P-FAK	Unstimulated	1	
	R1881	2,2	0,1
	R1881 + 6	0,8	0,03
paxillin	Unstimulated	1	
	R1881	1	0,02
	R1881 + 6	1	0,03
	6	1,1	0,03
P-paxillin	Unstimulated	1	
	R1881	3,1	0,02
	R1881 + 6	1	0,04
	6	0,9	0,05
ERK	Unstimulated	1	
	R1881	1	0,04
	R1881 + 6	0,9	0,06
	6	0,9	0,06
P-ERK	Unstimulated	1	
	R1881	4,1	0,3
	R1881 + 6	1,2	0,03
	6	1	0,06
P-RSK	Unstimulated	1	
	R1881	2,6	0,3
	R1881 + 4	0,2	0,03
	4	0,2	0,04
tubulin	Unstimulated	1	
	R1881	1	0,03
	R1881 + 6	1	0,05
	6	1,1	0,03

Quantification of Western blots presented in Figure 6c

		fold increase over the basal level n=3	SD
FAK	Unstimulated	1	
	R1881	0,9	0,04
	R1881 + Enz	1	0,02
	R1881 + 4	1	0,04
	R1881 + 6	1,1	0,03
P-FAK	Unstimulated	1	
	R1881	2	0,1
	R1881 + Enz	0,5	0,09
	R1881 + 4	0,5	0,09
	R1881 + 6	0,5	0,09
paxillin	Unstimulated	1	
	R1881	1	0,02
	R1881 + Enz	1	0,03
	R1881 + 4	1	0,03
	R1881 + 6	1	0,02
P-paxillin	Unstimulated	1	
	R1881	2,7	0,3
	R1881 + Enz	0,7	0,05
	R1881 + 4	0,8	0,04
	R1881 + 6	0,6	0,05
ERK	Unstimulated	1	
	R1881	1,1	0,04
	R1881 + Enz	1	0,06
	R1881 + 4	0,9	0,06
	R1881 + 6	1	0,02
P-ERK	Unstimulated	1	
	R1881	3,7	0,4
	R1881 + Enz	1	0,06
	R1881 + 4	0,7	0,06
	R1881 + 6	0,8	0,06
P-RSK	Unstimulated	1	
	R1881	3	0,2
	R1881 + Enz	0,2	0,03
	R1881 + 4	0,4	0,04
	R1881 + 6	0,1	0,03
tubulin	Unstimulated	1	

	R1881	1	0,06
	R1881 + Enz	1,1	0,07
	R1881 + 4	1,1	0,2
	R1881 + 6	1,1	0,03
P-Src	Unstimulated	1	
	R1881	2,1	0,2
	R1881 + Enz	0,9	0,04
	R1881 + 4	0,9	0,04
	R1881 + 6	0,8	0,05
Src	Unstimulated	1	
	R1881	0,9	0,02
	R1881 + Enz	0,9	0,03
	R1881 + 4	1	0,02
	R1881 + 6	1	0,02

P-Akt	Unstimulated	1	
	R1881	0,6	0,02
	R1881 + Enz	0,6	0,04
	R1881 + 4	0,6	0,05
	R1881 + 6	0,8	0,04
Akt	Unstimulated	1	
	R1881	1,1	0,03
	R1881 + Enz	1,1	0,04
	R1881 + 4	1,1	0,03
	R1881 + 6	1	0,05

Figure 6 d

Upper panels: lysates

AR

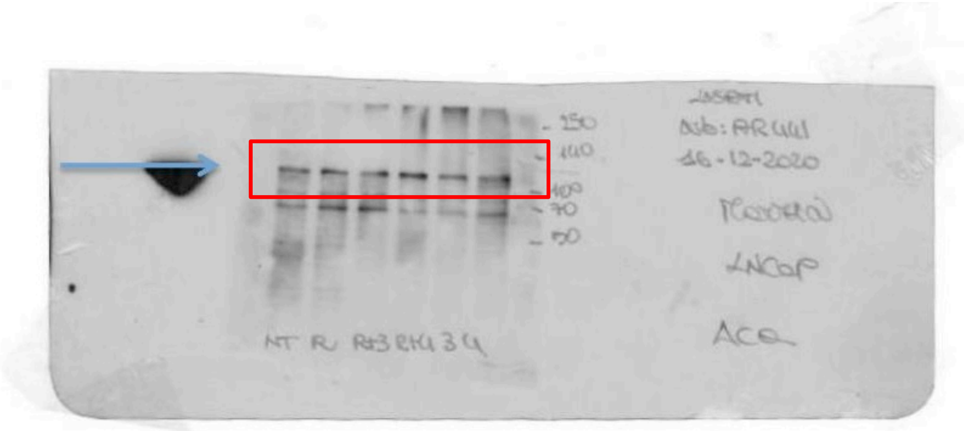

TRPM8

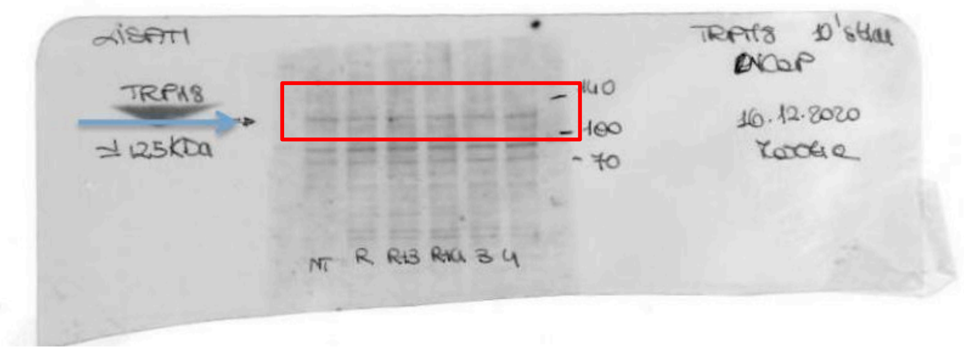

Quantification of Western blots presented in Figure 6d upper panel

		Average (n=3)	SD
AR	Unstimulated	1	
	R1881	1	0,04
	R1881 + 4	0,9	0,06
	R1881 + 6	1,1	0,06
	4	0,9	0,06
	6	0,9	0,02
TRPM8	Unstimulated	1	

	R1881	0,9	0,2
	R1881 + 4	1	0,03
	R1881 + 6	1	0,04
	4	1	0,03
	6	1,1	0,04

Quantification of Western blots presented in Figure 6d lower panel

		**Average (n=3)**	**SD**
AR	Unstimulated	1	
	R1881	1	0,03
	R1881 + 4	1,1	0,04
	R1881 + 6	1	0,04
	4	1,1	0,05
	6	1,1	0,07
TRPM8	Unstimulated	1	
	R1881	2,9	0,02
	R1881 + 4	0,7	0,01
	R1881 + 6	0,6	0,01
	4	0,3	0,01
	6	0,3	0,01

Lower panels: IP anti-AR

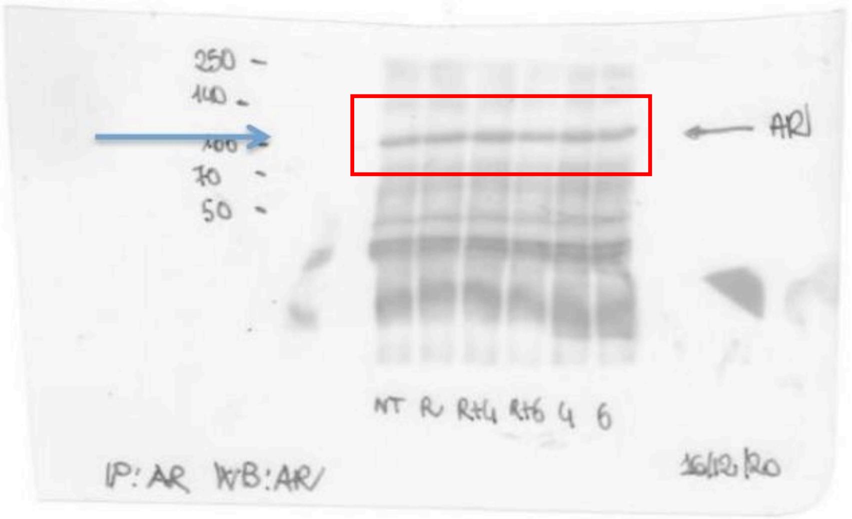

AR

TRPM8

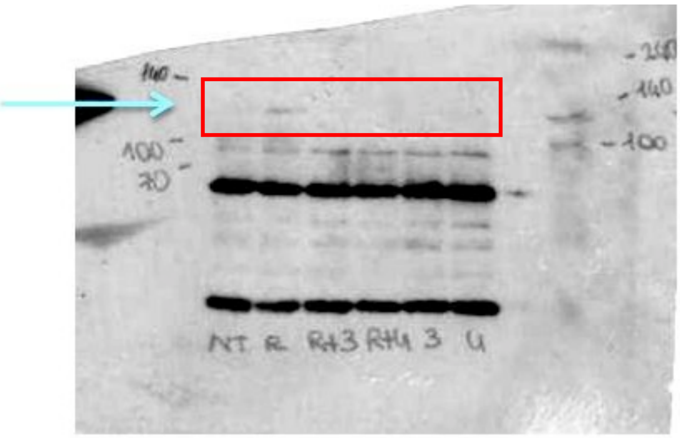

Middle panels: ctrl IgG

AR

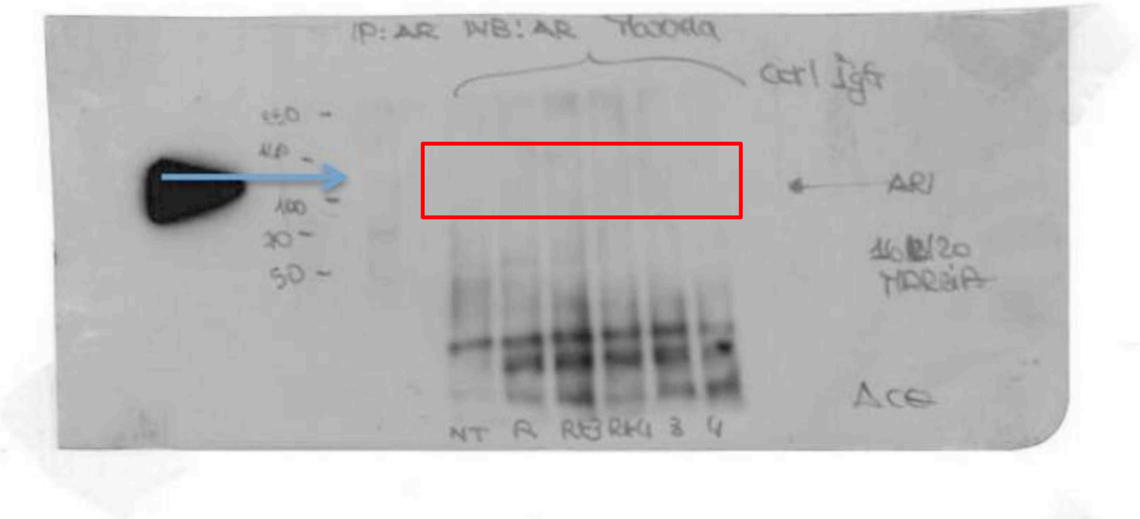

TRPM8

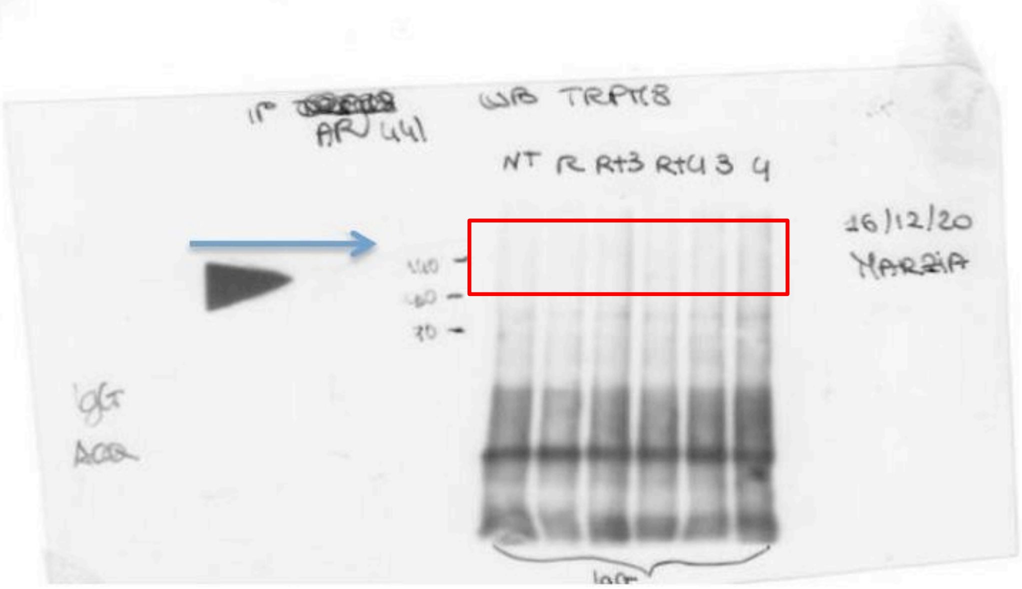

Figure 6 panel e

AR

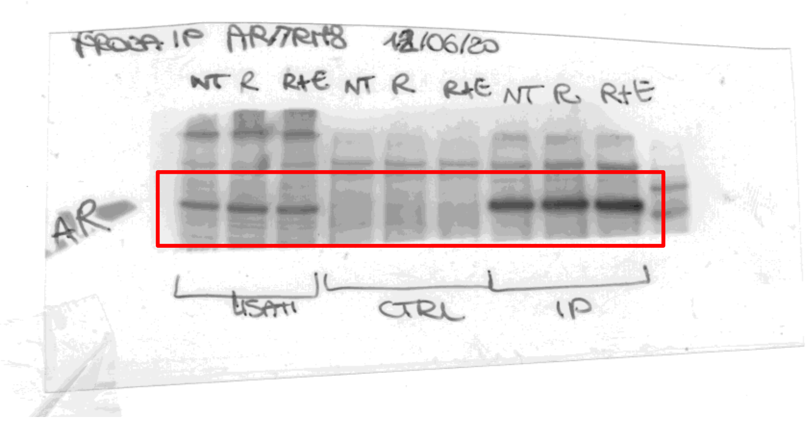

TRPM8

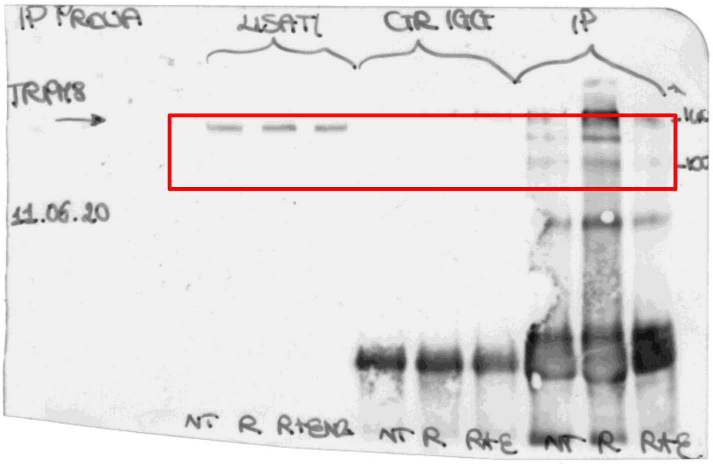

Quantification of Western blots presented in Figure 6e

		Average (n=3)	SD
AR	Unstimulated	1	
	R1881	1	0,03
	R1881+enz	1,1	0,02
	Unstimulated	0,1	0,09
	R1881	0,1	0,03
	R1881+enz	0,1	0,05
	Unstimulated	1	

	R1881	1	0,02
	R1881+enz	1	0,01
	Unstimulated	1	
	R1881	1	0,03
	R1881+enz	1,1	0,01
TRPM8	Unstimulated	1	
	R1881	1	0,05
	R1881+enz	1,1	0,05
	Unstimulated	0,1	0,05
	R1881	0,1	0,02
	R1881+enz	0,1	0,02
	Unstimulated	0	0
	R1881	0	0
	R1881+enz	0	0
	Unstimulated	1	
	R1881	3,1	0,04
	R1881+enz	0,3	0,01

Figure S5 panel a

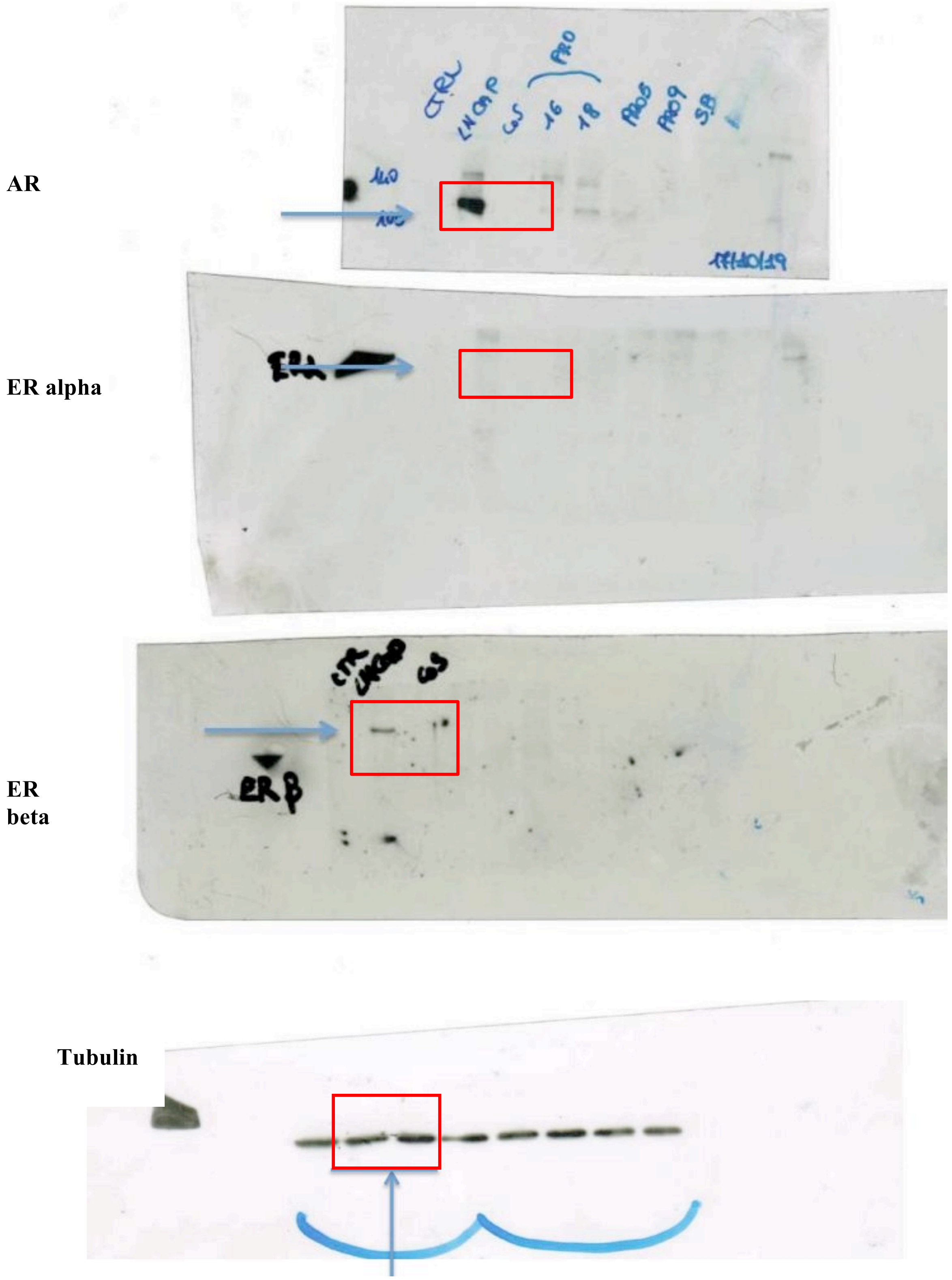

Figure S5 panel b

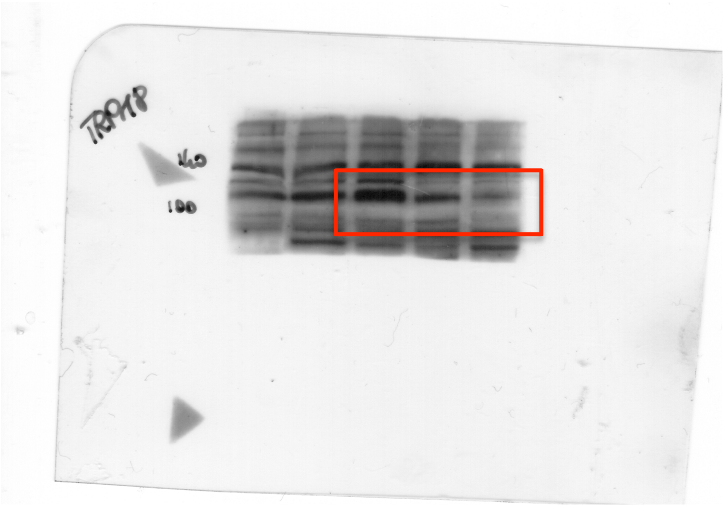

TRPM8

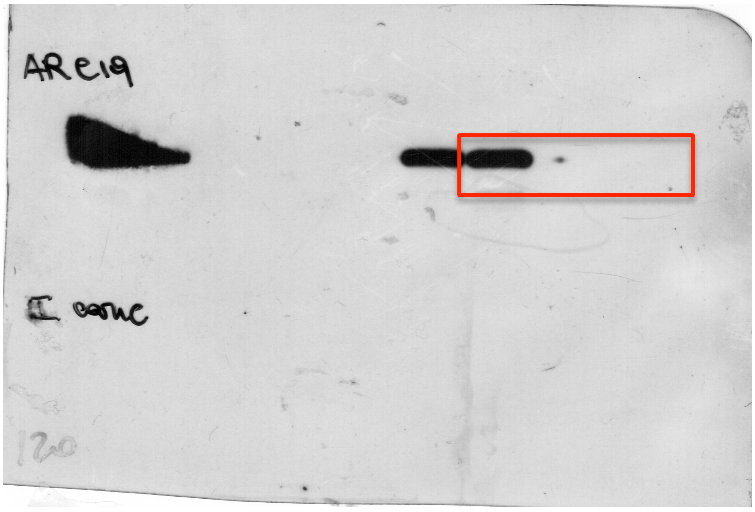

AR

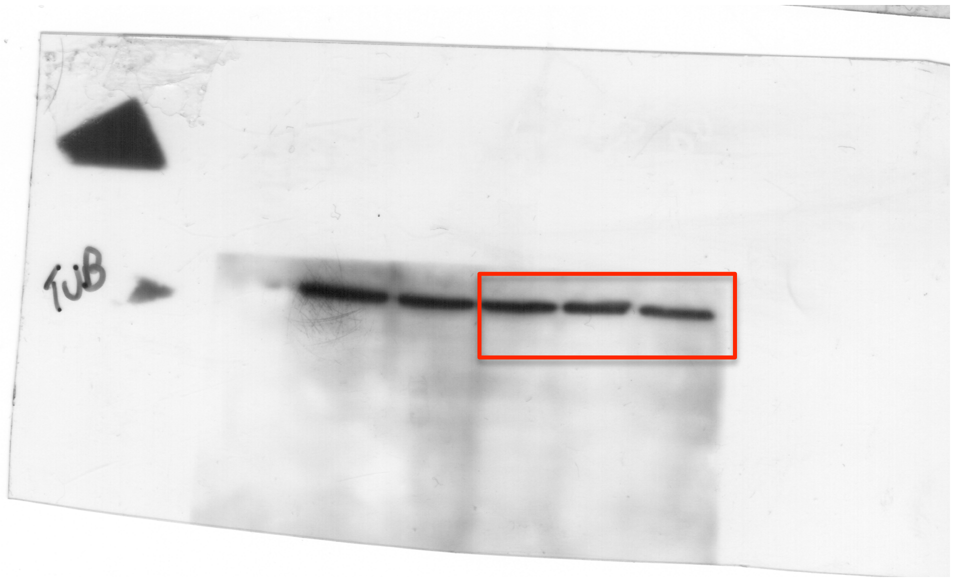

Tubulin

Quantification of Western blots presented in Figure S5 b

		Average (n=3)	SD
TRPM8/ Tubulin ratio	LNCaP	3	0,05
	PC3	2,7	0,08
	DU145	1,3	0,07

AR/ Tubulin ratio	LNCaP	3	0,05
	PC3	0	0
	DU145	0	0

Figure S6 panel a

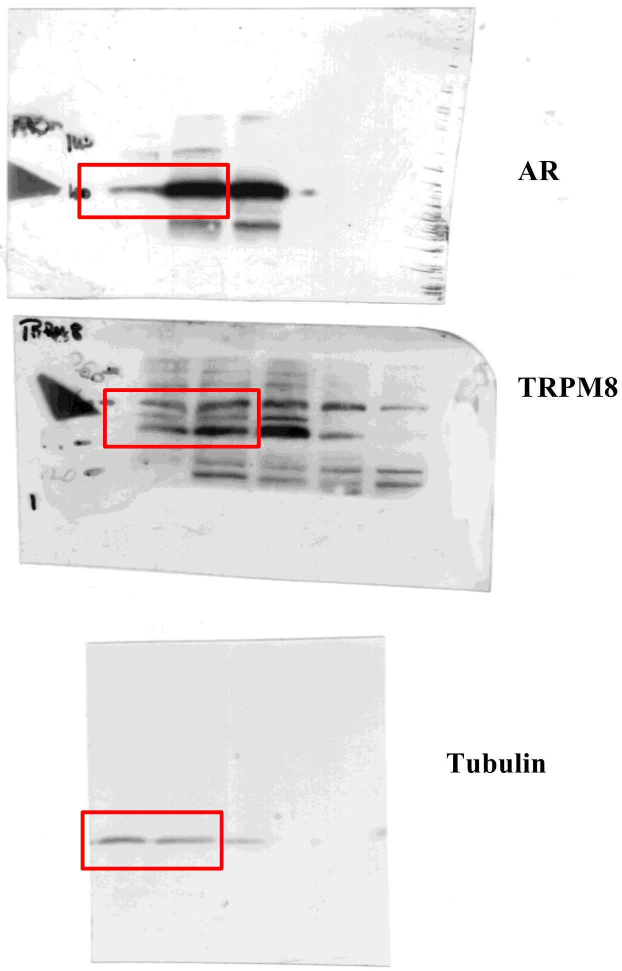

Quantification of Western blots presented in Figure S6 a

		Average (n=3)	SD
AR/Tubulin ratio	PNT-2	1	
	LNCaP	4,1	0,03
TRPM8/Tubulin Ratio	PNT-2	1	
	LNCaP	2,3	0,09
